# Mesenchymal Stem Cell Therapy in Multiple Sclerosis: A Systematic Review and Meta-Analysis

**DOI:** 10.3390/jcm12196311

**Published:** 2023-09-30

**Authors:** Md Asiful Islam, Sayeda Sadia Alam, Shoumik Kundu, Saleh Ahmed, Shabiha Sultana, Azim Patar, Tareq Hossan

**Affiliations:** 1 WHO Collaborating Centre for Global Women’s Health, Institute of Metabolism and Systems Research, College of Medical and Dental Sciences, University of Birmingham, Birmingham B15 2TT, UK; 2 Department of Biochemistry and Molecular Biology, Faculty of Biological Sciences, Jahangirnagar University, Savar, Dhaka 1342, Bangladesh; 3 Department of Chemistry and Biochemistry, Texas Tech University, 2500 Broadway St, Lubbock, TX 79409, USA; shkundu@ttu.edu; 4 Center for Biotechnology and Genomic Medicine, Medical College of Georgia, Augusta University, Augusta, GA 30912, USA; 5 Department of Cellular Biology and Anatomy, Medical College of Georgia, Augusta University, Augusta, GA 30912, USA; 6 Department of Neuroscience, School of Medical Sciences, Universiti Sains Malaysia, Kubang Kerian 16150, Kelantan, Malaysia; azimpatar@usm.my; 7 Department of Internal Medicine, Division of Oncology, Washington University School of Medicine in St. Louis, St. Louis, MO 63110, USA

**Keywords:** multiple sclerosis, mesenchymal stem cells, efficacy, expanded disability status scale, safety, systematic review, meta-analysis

## Abstract

The assurance of safety and effectiveness is a significant focal point in all therapeutic approaches. Although mesenchymal stem cells (MSCs) have been identified as a potential novel therapeutic strategy for multiple sclerosis (MS), existing evidence regarding the effectiveness and safety of this strategy remains inconclusive. Thus, the primary aim of this systematic review and meta-analysis (SRMA) was to comprehensively assess the effectiveness and safety of MSC therapy in individuals diagnosed with MS. A comprehensive search was conducted using appropriate keywords in the PubMed, Scopus, Cochrane, ScienceDirect, and Google Scholar databases to determine the eligible studies. The change in the expanded disability status scale (EDSS) score from baseline to follow-up was used to assess MSC efficacy. The effectiveness of the therapy was assessed using a random-effects model, which calculated the combined prevalence and 95% confidence intervals (CIs) for MS patients who experienced improvement, stability, or worsening of their condition. The protocol was registered in PROSPERO (CRD42020209671). The findings indicate that 40.4% (95% CI: 30.6–50.2) of MS patients exhibited improvements following MSC therapy, 32.8% (95% CI: 25.5–40.1) remained stable, and 18.1% (95% CI: 12.0–24.2) experienced a worsening of their condition. Although no major complications were observed, headaches 57.6 [37.9–77.3] and fever 53.1 [20.7–85.4] were commonly reported as minor adverse events. All of the results reported in this meta-analysis are consistent and credible according to the sensitivity analyses. Regardless of different individual studies, our meta-analysis provides a comprehensive overview showing the potential of MSC therapy as a possible effective treatment strategy for patients with MS.

## 1. Introduction

Multiple sclerosis (MS) is a pathological condition affecting the central nervous system (CNS) characterised by an autoimmune response resulting in inflammation, demyelination, and degeneration of axons. The majority of individuals diagnosed with MS exhibit a disease course characterised by periods of relapse and remission, which can persist over an extended duration. Primary progressive multiple sclerosis (PPMS) is a condition that impacts approximately 20% of individuals diagnosed with MS. It is distinguished by a progressive decline in neurological function from the initial manifestation of symptoms, without the occurrence of early relapses or remissions. [1,2]. Secondary progressive multiple sclerosis (SPMS) refers to a subtype of multiple sclerosis characterised by a gradual and continuous progression of symptoms, with or without remission. It occurs approximately 10 to 20 years after the initial onset of the disease. The primary characteristic symptoms encompass difficulties related to mobility and gait [3]. In MS patients, the disabilities in mobility and gait are quantified using the expanded disability status scale (EDSS), which is widely recognised as the predominant scale employed in clinical trials focusing on MS over the course of the follow-up [4].

Pertaining to various rates of disease progression, there is no definitive treatment for MS at this time. Current therapeutic approaches address the objective of shortening the duration of recovery following an attack, mitigating the progression of the disease, and attenuating the symptoms associated with multiple sclerosis. [3,5]. In the absence of timely intervention, the immune system initiates an attack on the myelin sheath, a protective covering, resulting in irreversible damage or degeneration of the nerves. It has been reported that patients receiving corticosteroid treatments, specifically prednisolone and intravenous prednisolone, in a clinical setting exhibit reduced nerve inflammation [6]. In cases where patients do not respond to steroids during the initial MS attacks, plasma exchange, also known as plasmapheresis, may be employed as an alternative treatment [7]. Ocrelizumab, commercially known as Ocrevus, is the only disease-modifying medication authorised by the U.S. Food and Drug Administration (FDA) for the treatment of primary progressive multiple sclerosis (PPMS) disease progression [8]. Injectable drug treatments such as interferon beta medications (to reduce the frequency and severity of relapses) and glatiramer acetate (to block the immune system from attacking the myelin sheath) may not be feasible options for relapsing or remitting disease progression or SPMS due to long-term side effects such as flu-like symptoms and skin irritation at the injection site [8]. There are several alternative treatment options available, such as oral medications like fingolimod, dimethyl fumarate, and diroximel fumarate, as well as infusion treatments like natalizumab and alemtuzumab. However, it is important to note that these treatments are associated with various side effects, including an increased risk of bacterial and viral infections [3]. Mesenchymal stem cells (MSCs) are stromal cells residing in many tissues including bone marrow, adipose tissues [9], and umbilical cord tissue [10,11]. MSCs have shown different magnitudes of effects on EDSS scores and magnetic resonance imaging (MRI) lesion outcomes reported in clinical trials. However, most trials were under-reported due to the low number of treated subjects, different dosages [3] used in the studies, the feasibility of autologous [12] or allogenic transplantation [3], and the unclear therapeutic window after the treatment effect. Several human clinical trials have reported a favorable safety profile on transplantation of these multipotent stem cells [13,14]. While the safety of using MSCs in the treatment of diseases such as hematological, cardiac, and inflammatory diseases has been extensively documented, there is limited research available regarding their application in the context of MS [14]. Hence, the utilization of MSCs as an alternative therapeutic approach for managing the progression of MS is garnering growing interest [15,16]. In addition to unique characteristics of MSCs, such as higher proliferation capacity and convenient availability, MSCs also consist of numerous cytokines, mediators, and signaling molecules. These substances play a crucial role in effectively regulating inflammatory responses and controlling the infiltration process, ultimately leading to a well-regulated process of tissue regeneration, healing, and repair [17]. Although other treatments including disease-modifying drugs (i.e., Ocrelizumab, Fingolimod, Teriflunomide etc.) [18,19,20] are available for the management of MS, they are not very effective in severe cases of MS. Additionally, they may also exhibit severe adverse effects. Therefore, emerging therapies such as MSC therapy have shown promising results in treating severe cases of MS. These innovative treatments aim to repair damaged nerve cells and halt disease progression, offering new hope for patients who have not responded well to conventional options.

In this meta-analysis, we sought to find out the feasibility, safety, and efficacy issues of using MSCs treatment, either intravenously [21] or intrathecally injected [22], in relation to the improvement of EDSS scores and MRI lesion outcomes among MS patients. The other measures of possible treatment effects are also reported. Therefore, we collected clinical trials pertaining to MS, encompassing both randomised and non-randomised studies, to evaluate the therapeutic impact or efficacy of MSCs on individuals with MS.

## 2. Materials and Methods

### 2.1. Systematic Review Protocol

This systematic review with a meta-analysis (SRMA) protocol was registered with PROSPERO (CRD42020209671) and carried out according to the PRISMA guidelines [23].

### 2.2. Eligibility Criteria

We only included studies in this SRMA that reported on the efficacy and safety of MSC therapy in human patients with MS based on the changes in the Expanded Disability Status Scale (EDSS) score from the baseline to follow-up period. We also included if the studies provided incidents of adverse events due to using MSCs. EDSS is the most popular and useful tool for measuring outcomes in MS patients. The scale has 20 steps, with the best score being 0 (a normal neurological test), the worst being 10 (MS-related mortality), and with 0.5 steps in between [24,25]. No restrictions were imposed on the language, time, and sex. Only clinical studies (both randomised and non-randomised) on human subjects (adults: aged 18 or above) were considered eligible. Meeting abstracts, review articles, case reports, non-human studies, theses, and opinions were excluded.

### 2.3. Search Strategy

Search strategies were developed to identify relevant articles in PubMed, Cochrane, Scopus, ScienceDirect, and Google Scholar databases utilizing appropriate keywords. The following keywords were searched across databases: multiple sclerosis, disseminated sclerosis, MS, mesenchymal, MSC, MSCs, and bone marrow stromal cells. Detailed search strategies were listed in Table S1. The last search was performed on 20 July 2023. To ensure a robust search, the reference lists of the retrieved reports were also searched to identify any additional publications that were relevant to the topic. EndNote X8 software was used to integrate the references, and before abstract evaluation, duplicate studies were identified and eliminated. The studies were independently searched and investigated by four authors (S.A., S.S.A., S.S., and S.K.). Disagreements about study eligibility and inclusion were resolved after consultation with M.A.I and A.P.

### 2.4. Data Extraction

Data extraction of the included studies was independently performed by four authors (S.A., S.S.A., S.S., and S.K.). Any discrepancies regarding data extraction were resolved with the help of another author (M.A.I.). The data and information extracted from the included studies covered various aspects, including the last name of the first author, the year of publication, the study design, the country of origin of the patients, the number and age of the participants, the duration of the disease, the types of MS, the sources of MSC, the follow-up period, the number of patients who experienced improvement, stability, or worsening, any adverse events reported, and the concluding remarks. The effectiveness of the treatment was assessed by examining the alterations in the Expanded Disability Status Scale (EDSS) score between the baseline and follow-up period. The post–treatment decline or increment of the EDSS score was regarded as an improvement or worsening of the disease condition, respectively. The patients were considered stable if no change of EDSS score was observed at the end of the follow-up period.

### 2.5. Quality Assessment

Using the critical evaluation tools offered by the Joanna Briggs Institute (JBI), two writers (S.S.A. and S.K.) evaluated the level of quality of the eligible studies. Based on total scores falling below 50%, between 50% and 70%, or above 70%, the studies were categorised as low-quality, moderate-quality, and high-quality [26]. The writers had discussions to settle any discrepancies. To assess the publication bias, a graphical representation known as a funnel plot was created to display the efficacy outcomes (improved, stable, and worsened) in patients with MS. The asymmetry of the funnel plot was subsequently confirmed using Egger’s test, with a significance level of *p* < 0.05 being considered statistically significant.

### 2.6. Determination of Safety and Efficacy

The safety and efficacy outcomes (improved, stable, and worsened) were calculated using a random-effects model with pooled prevalence and 95% confidence intervals (CIs) in MS patients. To quantify heterogeneity, the *I*^2^ statistic and Cochran’s Q test were employed, with *I*^2^ more than 75%, between 50 and 70%, and less than 50% indicating considerable, moderate, and low heterogeneity, respectively, with *p* < 0.05 being considered statistically significant. The metaprop codes available in the meta (version 4.11–0) and metafor (version 2.4–0) packages of R (version 3.6.3) and RStudio (version 1.3.1093) were utilised to perform all of the statistical analyses and generate the plots [27].

### 2.7. Subgroup and Sensitivity Analysis

In subgroup analysis, we estimated the efficacy of stem cell therapy based on (i) follow-up period, (ii) source of the MSCs, and (iii) mode of MSCs administration. Sensitivity analyses were carried out using the following methods to explore the sources of heterogeneity and verify the findings’ robustness: (i) eliminating studies of poor quality (high risk of bias) and (ii) omitting studies with a small sample size (n < 10). If an adverse event was reported in more than one study, we only considered that in the sensitivity analysis.

## 3. Results

### 3.1. Study Selection and Characteristics

**Figure 1:**
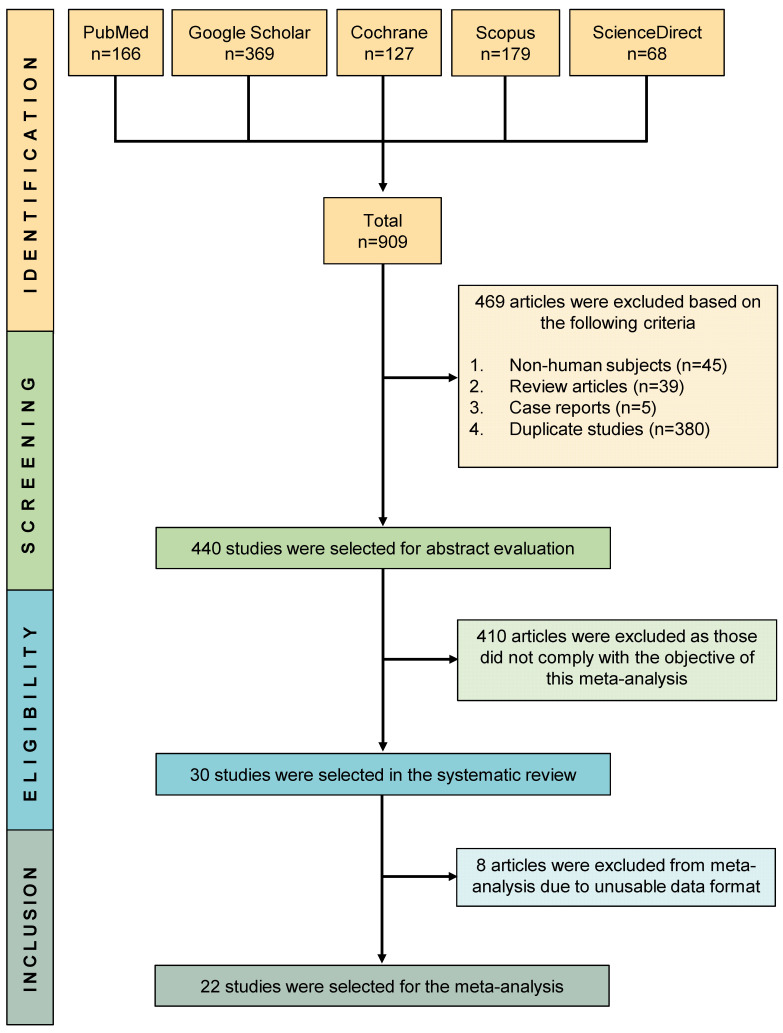
PRISMA flow diagram of study selection.

Searches in different databases such as PubMed, Scopus, Cochrane, ScienceDirect, and Google Scholar resulted in a total of 909 studies, from which 440 studies were screened following the removal of 469 studies (non–human subjects = 45, review articles = 39, case reports = 5, and duplicate studies = 380). Finally, a total of 30 studies were incorporated into the systematic review, while 22 studies were included in the subsequent meta-analysis. (Figure 1). Table 1 presents a comprehensive overview of the specific characteristics and references of the included studies.

### 3.2. Safety and Efficacy

**Figure 2:**
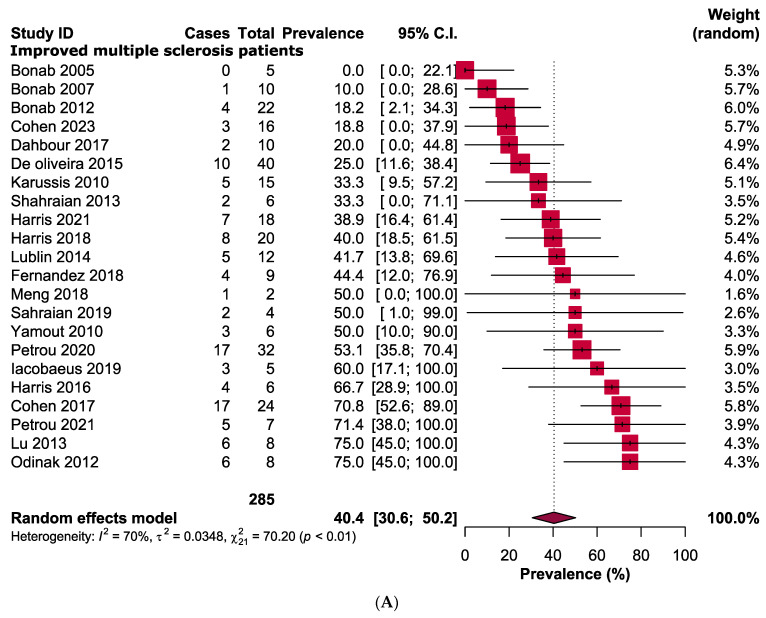

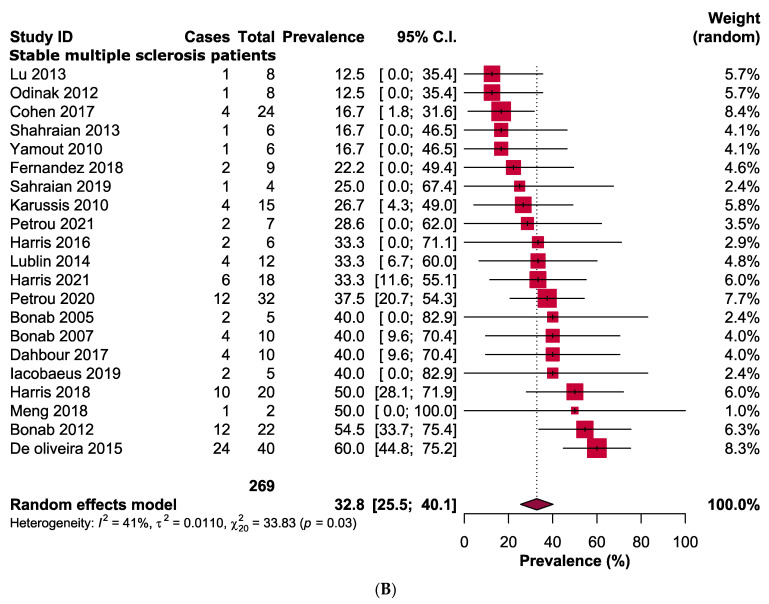

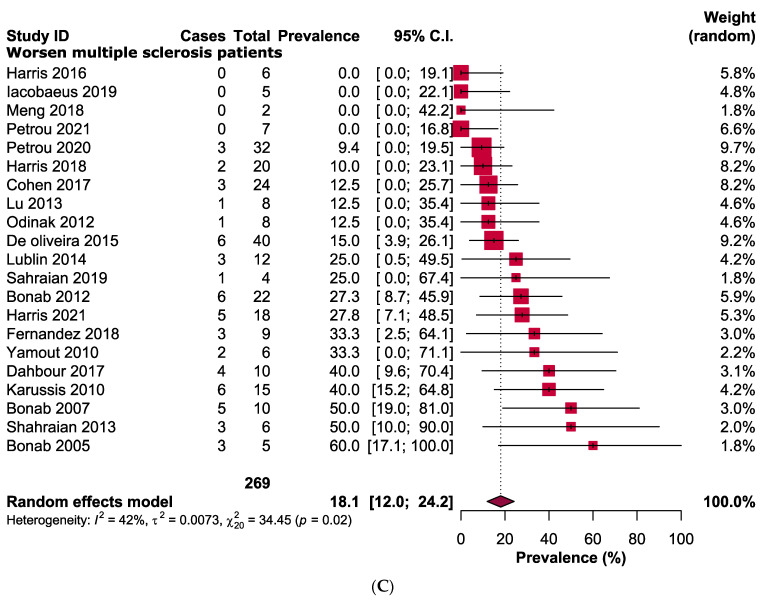
Forest plots representing the pooled prevalence of (**A**) improved, (**B**) stable, and (**C**) worsened patients with multiple sclerosis following mesenchymal stem cell therapy.

Following the MSCs therapy, it was observed that 40.4% [95% CI: 30.6–50.2] of the patients with MS experienced improvement. Additionally, 32.8% [95% CI: 25.5–40.1] of the patients remained stable while 18.1% [95% CI: 12.0–24.2] experienced a worsening of their condition, as indicated by changes in their EDSS score (Figure 2). Regarding the safety of MSCs therapy, headache 57.6% [95% CI: 37.9–77.3], fever 53.1% [95% CI: 20.7–85.4], urinary tract infections 23.9% [95% CI: 9.5–38.3], and respiratory tract infections 7.9 [0.7–15.1] were the most commonly reported adverse events, while no major complications were observed (Table 2 and Figure S1).

**Figure 3:**
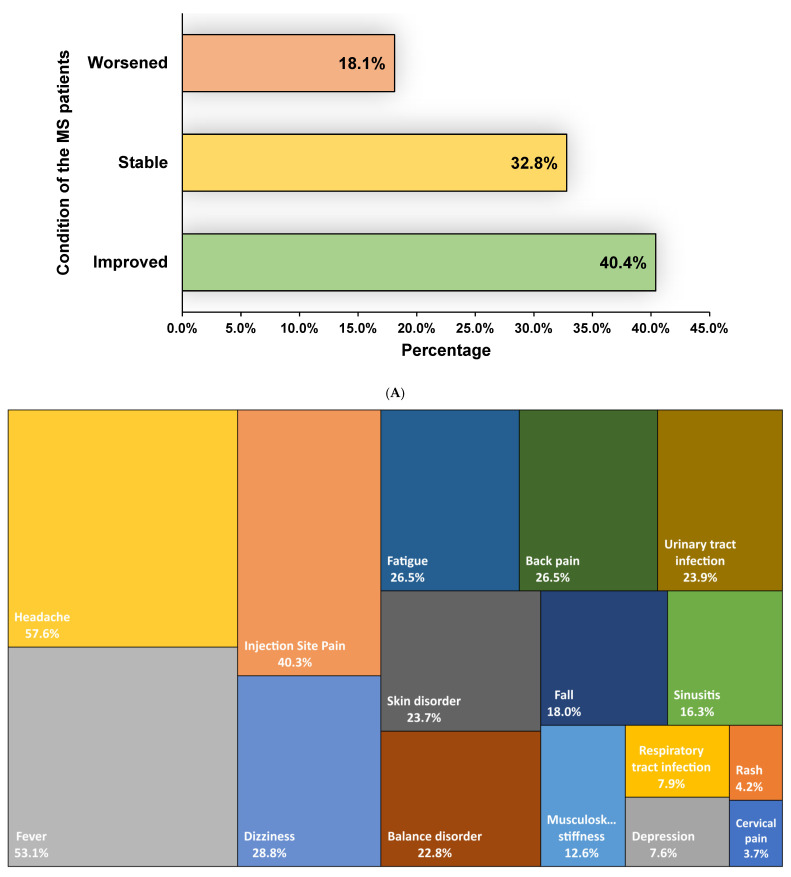
Summary findings of (**A**) effectiveness and (**B**) adverse events observed in patients with multiple sclerosis following mesenchymal stem cell therapy.

Interestingly, short-term follow-ups (≤6 months) seemed to be more efficacious, as 45.8% [95% CI: 20.2–71.5] of the MS patients improved, and 35.6% [95% CI: 11.2–60.0] were stable. Similar results were observed in long-term follow-up (>12 months) as well, where 48.0% [95% CI: 31.3–64.7] of the patients improved and 29.9% [95% CI: 20.8–39.0] were stable (Table 3 and Figure S2). Mesenchymal stem cells (MSCs) from the umbilical cord or placenta appeared to be more efficient in comparison to bone-marrow-derived MSCs (improved: 56.7% vs. 38.5%, stable: 23% vs. 34.1% and worsened: 15.8% vs. 18.4%). In terms of the mode of MSCs administration, intravenous administration was more efficacious in comparison to intrathecal administration (improved: 57.6% [95% CI: 44.1–71.0], *I*^2^ = 35% vs. 32.8 [95% CI: 21.6–44.0], *I*^2^ = 63%) (Table 3 and Figure S2). Consolidated data on the safety and efficacy of MSCs in MS is reported in Figure 3.

### 3.3. Publication Bias Assessment

**Figure 4:**
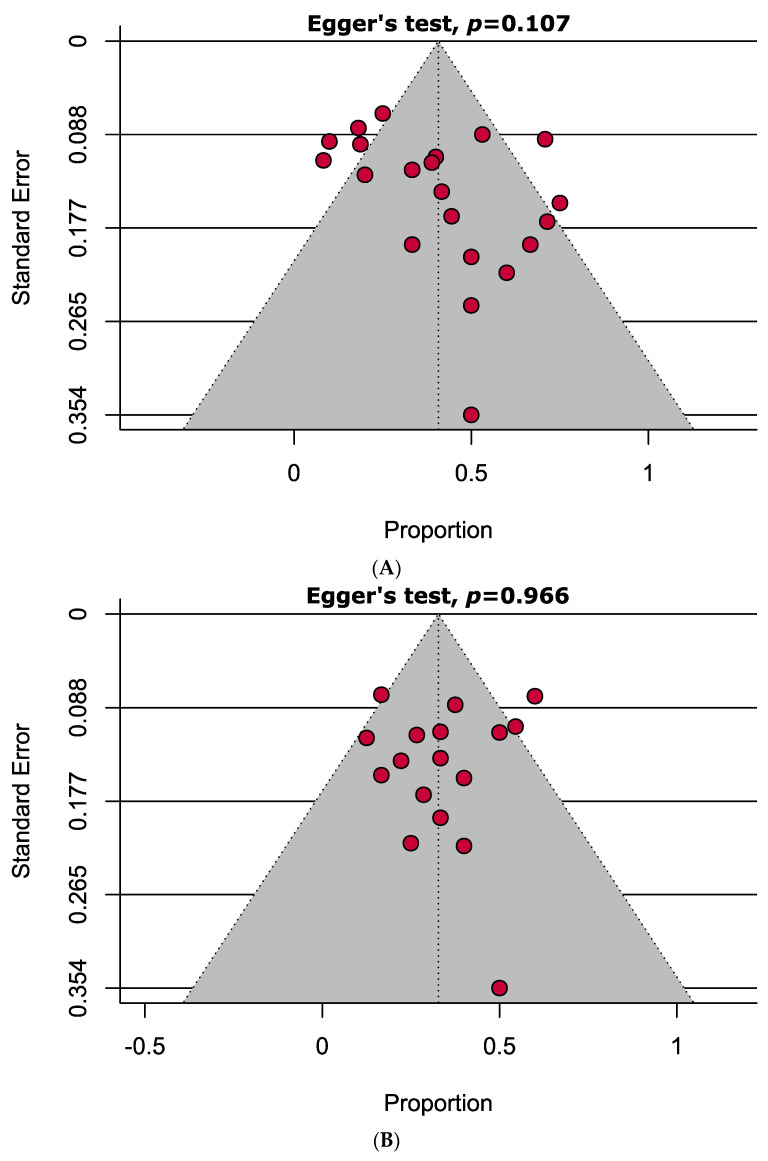

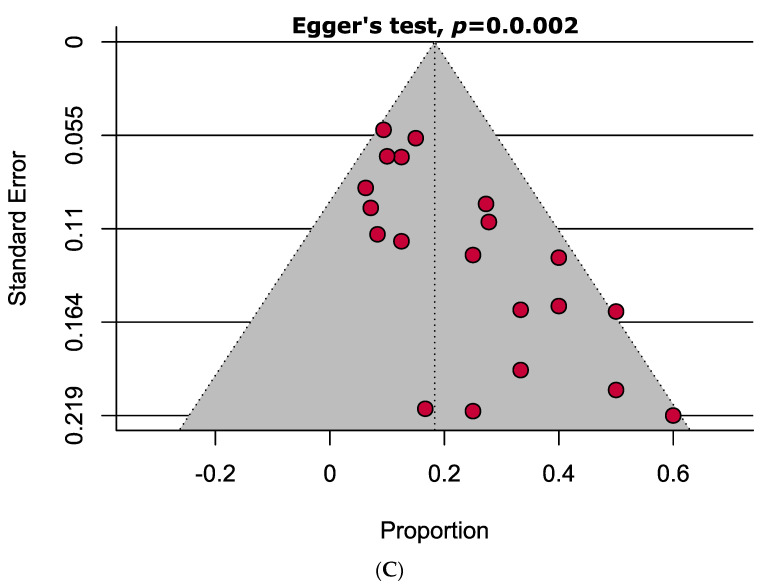
Funnel plots representing no publication bias in the (**A**) improved and (**B**) stable group; however, publication bias is present in the (**C**) worsened group.

According to the JBI critical appraisal tools, half of the studies were considered to be moderate quality, while the remaining studies were classified as high quality. Notably, no studies were identified as being of low quality (Tables S2 and S3). The examination of the funnel plot and the implementation of Egger’s test revealed the absence of substantial publication bias in the improved and stable group. However, a significant presence of bias was observed in the worsening group (Figure 4).

### 3.4. Sensitivity Analysis

The results obtained from sensitivity analyses that excluded low-quality and small studies showed negligible changes in comparison to the main findings. (Table 4 and Figure S3). Based on our sensitivity tests, all of the findings in this meta-analysis are consistent and credible.

## 4. Discussion

The utilisation of stem cells and their derived products has gained significant interest within the field of regenerative medicine, primarily owing to their remarkable capacity to facilitate the restoration of damaged or diseased tissue in individuals afflicted with various medical conditions. A number of the stem cells, including embryonic stem cells, adult stem cells, and perinatal stem cells have been showing their capacity to regenerate specific cells in several neurodegenerative diseases such as Parkinson’s disease, Alzheimer’s disease, spinal cord injuries, amyotrophic lateral sclerosis, type 1 diabetes, heart disease, stroke, burns, cancer, and osteoarthritis [51,52]. Research has been carried out in animal models as well as humans to examine their functionality in the restoration of tissues or organs. A comprehensive and contemporary systematic review and meta-analysis could collate extensive datasets to evaluate the overall efficacy of stem-cell-based therapy in treating a specific disease. MS is a pathological condition of the central nervous system where the immune system attacks the protective myelin sheath of the neurons, leading to the impairment of signalling between the brain and body [53]. Permanent damage of the nerves was also reported in MS [54]. Stem-cells-based therapy could be a promising candidate for the treatment of MS. Interestingly, several studies have been conducted in humans to examine the possibilities of stem cell therapy in MS.

This systematic review and meta-analysis aimed to evaluate the efficacy and safety of MSCs therapy in patients diagnosed with MS, focusing on the EDSS score as the primary outcome measure. We observed that 40.4% (95% CI: 30.6–50.2) of MS patients demonstrated improvements, while 32.8% (95% CI: 25.5–40.1) remained stable, and 18.1% (95% CI: 12.0–24.2) experienced a deterioration in their condition after receiving MSCs therapy. The findings of this analysis unveiled a potentially favourable impact of MSCs therapy for MS. However, the outcome depends on several factors, including age, the onset, and severity of the disease. Moreover, the origins of MSCs, specifically whether they are derived from a young or aged donor, as well as the source of collection (such as bone marrow, adipose tissue, or umbilical cord tissue) may also have an impact on the therapeutic results. Remarkably, our study revealed that MSCs derived from the umbilical cord or placenta exhibited greater efficacy in comparison to MSCs derived from bone marrow. Specifically, the improvement rates were 56.7% for umbilical cord or placental MSCs, whereas bone marrow derived MSCs showed an improvement rate of 38.5%. This finding is supported by several studies that have examined the comparative efficiency of MSCs generated from bone marrow, umbilical cord, or placenta [47,55,56]. There exist several potential factors that could contribute to the enhanced efficacy of umbilical cord or placenta-derived MSCs in comparison to bone-marrow-derived MSCs. For example, it has been observed that placenta or umbilical-cord-derived MSCs exhibit lower immunogenicity compared to bone-marrow-derived MSCs, indicating a reduced likelihood of rejection by the immune system of the recipient [55]. Furthermore, higher proliferation and differentiation capacities, differential gene expression patterns, as well as the noninvasive characteristics of umbilical cord or placenta MSCs may also play a role in higher efficacy. These findings suggest that the source of MSCs may play a crucial role in determining their therapeutic potential. Further research is needed to understand the underlying mechanisms behind this difference and explore the full potential of umbilical cord or placenta-derived MSCs in regenerative medicine. Additionally, the stability rates were 23% for umbilical cord or placental MSCs, while bone-marrow-derived MSCs exhibited a stability rate of 34.1%. Furthermore, the worsening rates were 15.8% for umbilical cord or placental MSCs, whereas bone marrow derived MSCs had a worsening rate of 18.4%. The effectiveness of the therapy may also be influenced by the route of administration. Based on our analysis, it is evident that the efficacy of intravenous administration was superior in comparison to intrathecal administration. The improvement observed for intravenous administration was 57.6% (95% CI: 44.1–71.0), with an *I^2^* value of 35%. On the other hand, intrathecal administration showed an improvement of 32.8% (95% CI: 21.6–44.0), with an *I^2^* value of 63%.

Current knowledge on the mechanisms of MSCs-driven therapy for MS indicate that it involves the modulation of a complex immunomodulatory pathway. In MS, oligodendrocyte apoptosis occurs due to an unknown mechanism. Activated microglia then phagocytose the apoptotic oligodendrocytes. Subsequently, the phagocytic cells activate the inflammatory immune response characterised by increased T helper cell 1 and 17 (Th1 and Th17), lymphocytes, and pro-inflammatory cytokines. The activity of the T helper cell 2 (Th2) and T regulatory cell (Treg) is suppressed. Together, these events lead to the demethylation and subsequent loss of axons [57,58]. Moreover, progressive MS is distinguished by persistent inflammation occurring inside an impermeable blood-brain barrier. It is accompanied by the activation of microglia and sustained participation of B cells and T cells. The occurrence of neurodegeneration is ultimately caused by the detrimental effects of reactive oxygen species (ROS) and nitrogen species (RNS) on both mitochondrial and axonal structures [59]. Studies have demonstrated that MSCs have the ability to modulate these processes by stimulating the production of anti-inflammatory cytokines, specifically by promoting the expansion of Th2 and Treg cells while concurrently inhibiting the activity of inflammatory cytokines by suppressing Th1 and Th17 lymphocytes. This intricate mechanism ultimately leads to the restoration of functional neurons [58]. As an adult stem cell, MSCs can be differentiated into a number of cell lineages, including neuronal cells [60]. Interestingly, MSCs therapy was reported to positively modulate the functions of astrocytes, oligodendrocytes, and neuronal axons [61]. It is likely that MSCs have the potential to initiate the regenerative processes necessary for the restoration of neuronal cells and supporting glial cells.

We also observed a few minor side effects of MSCs therapy to MS, including fever, headache, urinary tract infection, and respiratory tract infection. However, future research could find a solution to alleviate the side effects and improve strategies for treating MS using MSCs. A recent study by Riordan et al. [47] reported no serious adverse events following umbilical-cord-derived MSCs treatment for MS. Subsiding the manageable side effects, our meta-analysis clearly shows the prognostic effects of MSCs therapy for MS. Early data from international clinical trials presented at the European Committee for Treatment and Research in Multiple Sclerosis meeting in September 2019 indicated MSCs therapy as a safe and effective treatment. Phase II/III clinical trials have been conducted in different countries to further evaluate the safety and efficacy of the MSCs-based therapy for MS. The outcome of those trials could be further analysed to better understand the safety and efficacy of mesenchymal stem-cells-based therapy in MS. The present clinical data do not suggest precise dosages of MSCs. Nevertheless, the dosages can vary based on the clinical presentation of the patients. Notably, our study did not indicate any potential threatening adverse events of MSCs therapy.

Our meta-analysis has several strengths. To the best of our current understanding, this is the first meta-analysis to comprehensively examine the efficacy and safety of MSCs therapy in MS patients. This meta-analysis included a large number of studies and therefore a large number of individuals, which resulted in more robust estimates. Since there was just one study with a substantial publication bias, it is unlikely that we overlooked studies that may have changed the results. The sensitivity analyses yielded results that were highly consistent with the primary findings, thereby indicating the robustness of the meta-analysis. In addition, it is noteworthy that half of the studies incorporated in the analysis exhibited a high level of methodological quality, indicating a low risk of bias. Conversely, the remaining studies were found to possess an intermediate level of quality. This combination of high- and intermediate-quality studies contributes to the overall reliability and credibility of the findings. The existence of significant degrees of heterogeneity is one of the main drawbacks of this meta-analysis. Even though we investigated the causes of heterogeneity using subgroup and sensitivity analyses, the variables included in the studies did not completely explain the sources of heterogeneity.

## 5. Conclusions

In conclusion, MSCs therapy seemed to be an efficacious therapeutic strategy in treating patients with MS, as a majority of patients either improved or remained stable based on the EDSS score. In addition, as no major adverse events were identified, it appeared to be a safe therapeutic strategy in treating MS patients. However, further research, development of new technology, optimisation of MSCs doses, and larger clinical trials are needed to fully evaluate its long-term effectiveness and safety profile. 

## Figures and Tables

**Table 1:** Major Characteristics of the Included Studies.

Study ID [References]	Study Design	Country	Total Participants (Female)	Age (Mean ± SD/Range) (Years)	Patient Enrolment Time	Disease Duration (Mean ± SD/Range) (Years)	Types of MS with Corresponding Number of Participants	Source of MSCs	Amount of Cell Infusion	Method of Cell Suspension Administration	Follow–Up Period	Summary of Findings
Nabavi 2023 [28]	Randomised controlled trial	Iran	21 (16)	35.29 ± 8.44	December 2011– May 2014	9.71 ± 3.18	RRMS: 14 SPMS: 5 PPMS: 2	Bone marrow	2 × 10^6^ cells/kg	Intravenous	18 months	Although efficacy findings were not notable based on EDSS score changes, no major adverse events were reported.
Cohen 2023 [29]	Clinical trial	USA	18 (10)	47.4 ± 9.6	March 2019–March 2021	17.7 ± 7.9	SPMS: 14 PPMS: 4	Bone marrow	5 mL, 100–125 million	Intrathecal	28 weeks	Based on changes in EDSS score, MSCs therapy increased efficacy with some minor adverse events in patients.
Tremblay 2022 [30]	Randomised controlled trial	Canada	20 (7)	37.6 ± 6.9 for early and 37.6 ± 5.1 for delayed group	NR	5.7 ± 2.9 for early and 6.6 ± 2.7 for delayed group	PPMS: 6 RRMS: 8 SPMS: 6	Bone marrow	1–2 × 10^6^ MSCs/Kg	Intravenous	48 weeks	MSCs therapy did not cause significant changes in the EDSS score, hence it does not improve neurophysiological and clinical outcomes in patients with MS.
Harris 2021 [31]	Clinical trial		20 (14)	49 (27–65)	2014–2016	19 (10–32)	PPMS: 16 SPMS: 4	Bone marrow	9.4 × 10^6^ cells	Intrathecal	2 years	39% of MS patients improved after MSCs therapy based on EDSS without serious adverse events.
Uccelli 2021 [32]	Randomised controlled trial	Austria, Canada, Denmark, France, Italy, Iran, Spain, Sweden, and the UK	144 (87)	39.9 ± 6.70	July 16, 2012–July 31, 2019	2–15	PPMS: 6 RRMS: 8 SPMS: 6	Bone marrow	1–2 × 10^6^ MSCs/Kg	Intravenous	48 weeks	No significant changes in EDSS score occurred between the early and delayed group of MS patients. However, several adverse events were observed in the patients.
Petrou 2021 [33]	Clinical trial	Israel	24 (12)	47.0 ± 9.22	NR	13.4 ± 6.6	SPMS: 22 PPMS: 2	Bone marrow	1 × 10^6^ MSCs/Kg	Intravenous and Intrathecal	4 years	EDSS score was shown to decline in the majority of the patients (71%), and rest of them were stable. Also, no serious adverse events were observed.
Petrou 2020 [34]	Randomised controlled trial	Israel	48 (20)	47.63 ± 9.72	Feb 2015–June 2018	12.70 ± 7.51	SPMS: 41 PPMS: 7	Bone marrow	1 × 10^6^ MSCs/Kg	Intravenous and Intrathecal	14 months	Following MSCs therapy, 53% and 38% of the MS patients were shown to be improved and stable, evidenced by the declining EDSS score.
Baldassari 2019 [35]	Clinical trial	USA	22 (16)	46.4 ± 5.2	Mar 2011–Apr 2013	12.4 ± 9.4	SPMS: 13 RRMS: 9	Bone marrow and adipose tissue	NR	Intravenous	6 months	Treatment with MSCs did not exhibit any significant alteration in the EDSS score among patients with MS.
Bonab 2005 [4]	Clinical trial	Iran	5 (3)	31.0 ± NR	NR	6.0–15.0	NR	Bone marrow	5.5 mL; 6.0 × 10^6^ cells	Intrathecal	7 months	Although most of the patients did not improve according to EDSS score, the treatment procedure was considered to be safe.
Bonab 2007 [36]	Clinical trial	Iran	10 (7)	33.0 ± 5.9	NR	3.0–21.0	SPMS: 8 PPMS: 2	Bone marrow	5.5 mL; 8.7 × 10^6^ cells	Intrathecal	13–26 months	Treatment with MSCs could not be demonstrated as an effective strategy as 50% of the patients exhibited an increased EDSS score when compared to baseline.
Bonab 2012 [37]	Clinical trial	Iran	22 (18)	18.0–50.0	Jan 2008–Aug 2010	≤2– ≥ 15	SPMS: 20 PRMS: 2	Bone marrow	10.0 mL; 29.5 × 10^6^ cells	Intrathecal	12 months	Administration was reported to be safe; however, almost all the patients exhibited fever. Most of the patients remained stable at the end of follow–up.
Llufriu 2014 [38]	Randomised controlled trial	Spain	9 (7)	36.8 ± 8.4	Nov 2010–June 2012	8.1 ± 2.15	All RRMS	Bone marrow	1.03 × 10^6^–2.16 × 10^6^ (mean = 1.87 × 10^6^) cells/kg	Intravenous	12 months	No significant changes occurred in EDSS score after MSCs therapy, but it was considered to be safe.
Cohen 2017 [21]	Clinical trial	USA	25 (17)	46.4 ± 5.2	NR	15.4 ± 9.0	SPMS: 14 RRMS: 10	Bone marrow	1.9 × 10^6^ cells/kg	Intravenous	6 months	Administration of MSCs showed a noteworthy efficacy (decline of EDSS in 71% of patients). Although 40% of the patients experienced some minor adverse events though the treatment procedure, it was overall well-tolerated.
Cornick 2012 [12]	Clinical trial	UK	10 (3)	48.8 ± 4.1	Nov 2007–Aug 2010	14.4 ± 7.9	All SPMS	Bone marrow	1·6 × 10⁶ cells/kg	Intravenous	10 months	Significant improvements were observed (*p* = 0.028) based on the EDSS score, and the treatment was safe except for some minor adverse events associated with infections.
Dahbour 2017 [39]	Clinical trial	Jordan	10 (4)	34.9 ± 9.5	NR	9.6 ± 2.9	NR	Bone marrow	18.3 mL; 110 × 10^6^ cells	Intrathecal	12 months	Treatment with MSCs did not lower the EDSS score of most of the patients; however, it was reported to be safe, and some minor adverse events were observed.
De Oliveira 2015 [40]	Clinical trial	Brazil	44 (30)	37.3 ± 9.4	NR	4.0–20.0	SPMS: 34 PPMS: 3 RRMS: 7	Bone marrow	NR	NR	6 months	EDSS score declines in one-fourth of the patients, and 60% remained stable.
Fernandez 2018 [9]	Randomised controlled trial	Spain	30 (21)	46.3 ± 8.9	NR	17.7 ± 7.4	All SPMS	Adipose tissue	Low dose: 1.0 × 10^6^ cells/kg high dose: 4.0 x 10^6^ cells/kg	Intravenous	12 months	No significant change was noticed in the mean EDSS level upon completion of the trial.
Harris 2016 [41]	Clinical trial	USA	6 (4)	28.0–64.0	2005–2007	7.0–27.0	SPMS: 4 PPMS: 2	Bone marrow	0.06 × 10^6^ cells–16.0 × 10^6^ cells	Intrathecal	7.4 years	Treatment with MSCs depicted an effective outcome, as 66.6% were improved and the rest were stable.
Harris 2018 [42]	Clinical trial	USA	20 (6)	27.0–65.0	NR	10.0–32.0	SPMS: 16 PPMS: 4	Bone marrow	9.4 × 10^6^ cells	Intrathecal	12 months	40% of the patients showed a declined EDSS score. Although overall the treatment was safe and well-tolerated, headache occurred in 85% of the patients.
Iacobaeus 2019 [43]	Clinical trial	Sweden	7 (6)	18.0–50.0	Oct 2012–Jan 2015	2.0–20.0	SPMS: 5 PPMS: 2	Bone marrow	1.0–2.0 × 10^6^ cells/kg	Intrathecal	48 weeks	60% of the patients improved, and the rest remained stable.
Karussis 2010 [22]	Clinical trial	Israel	15 (8)	35.3 ± 8.6	NR	10.7 ± 2.9	NR	Bone marrow	63.2 ± 2.5 × 10^6^ cells	Intrathecal	6 months	EDSS score declined significantly; however, 66.6% of the participants suffered from fever and headache.
Li 2014 [10]	Randomised controlled trial	China	13 (9)	41.7 ± 5.6	Jan 2010–Dec 2012	2.9 ± 0.9	NR	Umbilical cord	4.0 × 10^6^ cells/kg	Intravenous	12 months	Marginal decrease of EDSS score was observed, indicating it as an efficacious strategy.
Lu 2013 [44]	Clinical trial	China	8 (6)	18.0–59.0	May 2010–Dec 2010	>4.0	All SPMS	Umbilical cord	Day 0: 40 mL, day 7, 14 and 21: 20 mL; 2.0 × 10^7^ cells	Intravenous	18 months	The treatment with MSCs was highly efficacious, and the EDSS scores of 75% of the patients decreased.
Lublin 2014 [45]	Randomised controlled trial	USA and Canada	16 (11)	18.0–65.0	NR	≥2.0	SPMS: 6 RRMS: 10	Placenta	240 mL; Low dose: 150.0 × 10^6^ cells, high dose: 600.0 × 10^6^ cells	Intravenous	12 months	This study exhibited a mixed outcome in terms of the EDSS score, although the rate of improvement was slightly satisfactory.
Meng 2018 [11]	Clinical trial	China	3 (1)	30.0–33.0	NR	5.0–9.0	SPMS: 2 RRMS: 1	Umbilical cord	1.0–2.0 × 10^6^ cells/kg.	Intravenous	10 years	With a prolonged follow-up period, 50% of the participants improved in case of EDSS score.
Odinak 2012 [46]	Clinical trial	Russia	8 (3)	24.0–47.0	NR	4.0–14.0	SPMS: 3 RRMS: 3 PPMS: 2	Bone marrow	2.0 × 10^6^ cells/kg.	Intravenous	12 months	Treatment with MSCs was highly efficacious, with 75% improvements and no notable adverse events.
Riordan 2018 [47]	Clinical trial	Panama	20 (12)	41.1 ± 9.2	Oct 2014–Feb 2015	7.7 ± NR	SPMS: 1 RRMS: 15 PPMS: 4	Umbilical cord	20.0 × 10^6^ cells/day	Intravenous	12 months	A mean decrease of 0.68 ± 1.49 was observed in the overall population.
Sahraian 2013 [48]	Clinical trial	Iran	10 (3)	28.0 ± 4.3	NR	3.0–16.0	All SPMS	Bone marrow	5.5 mL; 7.5 × 10^6^ cells	Intrathecal	5 years	Treatment with MSCs was not highly efficacious, as there was a mixture of improvement and worsening of the disease condition.
Sahraian 2019 [49]	Clinical trial	Iran	4 (1)	26.0–31.0	NR	5.0–10.0	SPMS: 3 RRMS:1	Bone marrow	57.0 × 10^6^ cells	Intrathecal	2 years	75% of the participants improved or remained stable following the MSC therapy, with no major adverse events.
Yamout 2010 [50]	Clinical trial	Lebanon	10 (6)	34.0–56.0	NR	11.0–31.0	SPMS: 9 RRMS: 1	Bone marrow	10.0 mL × 10^6^ cells	Intrathecal	12 months	Treatment with MSCs was efficacious, and the EDSS score declined for half of the patients. The treatment procedure was also reported to be safe.

SPMS: Secondary progressive multiple sclerosis, PPMS: Primary progressive multiple sclerosis, RRMS: Relapsing remitting multiple sclerosis, PRMS: Progressive relapsing multiple sclerosis, MSCs: Mesenchymal stem cells, EDSS: Expanded disability scale score, SD: Standard deviation, MS: Multiple sclerosis, NR: Not reported.

**Table 2:** Pooled prevalence of adverse events in patients with multiple sclerosis following mesenchymal stem cell therapy.

Adverse Events	Adverse Events [95% CIs] (%)	Number of Studies Analysed	Total Number of Multiple Sclerosis Patients	Heterogeneity	
				*I^2^*	*p*–Value
Headache	57.6 [37.9–77.3]	15	236	94%	<0.01
Fever	53.1 [20.7–85.4]	10	146	98%	<0.01
Urinary tract infection	23.9 [9.5–38.3]	7	132	81%	<0.01
Respiratory tract infection	7.9 [0.7–15.1]	5	94	41%	0.15
Dizziness	28.8 [5.6–51.9]	4	64	84%	<0.01
Fatigue	26.5 [0.0–54.3]	4	91	94%	<0.01
Skin disorder	23.7 [1.0–46.3]	4	55	85%	<0.01
Back pain	26.5 [1.5–51.5]	5	104	93%	<0.01
Balance disorder	22.8 [9.7–36.0]	2	39	0%	0.68
Depression	7.6 [0.0–15.3]	2	44	0%	0.37
Fall	18.0 [6.7–29.2]	4	79	38%	0.18
Rash	4.2 [0.0–9.9]	3	47	0%	0.53
Musculoskeletal stiffness	12.6 [3.9–21.4]	3	55	0%	0.97
Sinusitis	16.3 [0.0–46.5]	2	37	56%	0.13
Cervical pain	3.7 [0.0–9.6]	2	38	0%	0.38
Injection site pain	40.3 [3.3–77.2]	2	33	82%	0.02

CIs: Confidence intervals; NA: Not applicable.

**Table 3:** Sub–group analyses.

Outcomes	Prevalence [95% CIs] (%)	Number of Studies Analysed	Total Number of Multiple Sclerosis Patients	Heterogeneity	
				*I^2^*	*p*–Value
**Follow-up: ≤6 months**					
Improved	45.8 [20.2–71.5]	4	84	82%	<0.01
Stable	35.6 [11.2–60.0]			82%	<0.01
Worsened	15.4 [3.9–26.8]			48%	0.12
**Follow-up: >6 to 12 months**					
Improved	31.5 [17.8–45.2]	9	108	65%	<0.01
Stable	34.9 [22.0–47.9]	8	92	47%	0.07
Worsened	22.8 [13.2–32.4]	8	92	23%	0.25
**Follow-up: >12 months**					
Improved	48.0 [31.3–64.7]	9	93	65%	<0.01
Stable	29.9 [20.8–39.0]			0%	0.79
Worsened	15.3 [4.5–26.0]			50%	0.04
**Bone-marrow-derived stem cells**					
Improved	38.5 [28.2–48.9]	19	263	71%	<0.01
Stable	34.1 [26.0–42.3]	18	247	47%	0.01
Worsened	18.4 [11.7–25.0]	18	247	48%	0.01
**Umbilical cord or placenta-derived stem cells**					
Improved	56.7 [33.3–80.1]	3	22	22%	0.28
Stable	23.0 [6.2–39.9]			0%	0.37
Worsened	15.8 [0.3–31.4]			0%	0.56
**Intravenous administration**					
Improved	57.6 [44.1–71.0]	7	79	35%	0.16
Stable	18.6 [9.2–28.0]	6	63	0%	0.76
Worsened	15.9 [7.9–23.9]	7	79	0%	0.83
**Intrathecal administration**					
Improved	32.8 [21.6–44.0]	14	159	63%	<0.01
Stable	37.4 [29.3–45.5]	12	127	0%	0.47
Worsened	22.3 [10.7–33.9]	12	125	70%	<0.01

CIs: Confidence intervals.

**Table 4:** Sensitivity analyses.

Strategies of Sensitivity Analyses	Efficacy [95% Cis] (%)	Difference of Pooled Prevalence Compared to the Main Result	Number of Studies Analysed	Total Number of Multiple Sclerosis Patients
**Excluding low-quality studies**				
Improved	40.4 [30.6–50.2]	Unchanged	22	285
Stable	32.8 [25.5–40.1]	Unchanged	21	269
Worsened	18.1 [12.0–24.2]	Unchanged	21	269
**Excluding small studies**				
Improved	33.5 [22.2–44.7]	6.9% lower	11	219
Stable	39.2 [29.2–49.3]	6.4% higher	10	203
Worsened	20.3 [13.0–27.6]	2.2% higher	10	203

CIs: Confidence intervals.

## Data Availability

The data presented in this study are available within the article and Supplementary Materials.

## Supplementary Materials

The following supporting information can be downloaded at: https://www.mdpi.com/article/10.3390/jcm12196311/s1, Figure S1: Adverse events in patients with multiple sclerosis following the mesenchymal stem cell therapy; Figure S2: Subgroup analyses; Figure S3: Sensitivity analyses.; Table S1: Search strategy; Table S2: Quality assessment of the non–randomised experimental studies and Table S3: Quality assessment of the included randomised–controlled trials.
