# Supplementary material for: Mesenchymal Stem Cell Therapy in Multiple Sclerosis: A Systematic Review and Meta-Analysis
Source: J Clin Med. 2023 Sep 30;12(19):6311. doi: 10.3390/jcm12196311 (PMC10573670; doi:10.3390/jcm12196311)
Supplement: Supplementary file 1 [file jcm-12-06311-s001.zip › jcm-2586486-supplementary.pdf]

Table S1. Search strategy.

Databases	Search terms
PubMed	(Multiple sclerosis[Title] OR disseminated sclerosis[Title] OR MS[Title]) AND (Mesenchymal[Title] OR MSC[Title] OR MSCs[Title] OR Bone Marrow Stromal Cells[Title])
Cochrane	Title Abstract keyword: (Multiple sclerosis OR disseminated sclerosis OR MS) AND (Mesenchymal OR MSC OR MSCs OR Bone Marrow Stromal Cells)
Scopus	TITLE("Multiple sclerosis" OR "disseminated sclerosis" OR MS) AND TITLE(Mesenchymal OR MSC OR MSCs OR "Bone Marrow Stromal Cells")
ScienceDirect	Title: ("Multiple sclerosis" OR "disseminated sclerosis" OR MS) AND (Mesenchymal OR MSC OR MSCs OR "Bone Marrow Stromal Cells")
Google Scholar	allintitle:("Multiple sclerosis" OR "disseminated sclerosis") (Mesenchymal OR MSC OR MSCs OR "Bone Marrow Stromal Cells")

Table S2. Quality assessment of the non-randomised experimental studies.

No	Study ID	1	2	3	4	5	6	7	8	9	Yes (%)
1	Baldassari 2019	Y	NA	U	Y	Y	Y	Y	Y	Y	87.5
2	Bonab 2005	Y	NA	NA	N	Y	Y	NA	Y	N	66.7
3	Bonab 2007	Y	NA	NA	N	Y	Y	NA	Y	N	66.7
4	Bonab 2012	Y	NA	NA	N	Y	N	NA	Y	N	50.0
5	Cohen 2017	Y	NA	NA	N	Y	N	NA	Y	Y	66.7
6	Cornick 2012	Y	NA	NA	N	Y	Y	NA	Y	Y	83.3
7	Dahbour 2017	Y	NA	NA	N	Y	Y	NA	Y	Y	83.3
8	De oliveira 2015	Y	NA	NA	Y	Y	N	Y	Y	Y	85.7
9	Harris 2016	Y	NA	NA	N	Y	Y	NA	Y	N	66.7
10	Harris 2018	Y	NA	NA	N	Y	Y	NA	Y	Y	83.3
11	Iacobaeus 2019	Y	NA	NA	N	Y	Y	NA	Y	Y	83.3
12	Karussis 2010	Y	NA	Y	N	Y	Y	Y	Y	Y	87.5
13	Lu 2013	Y	NA	NA	N	Y	Y	NA	Y	N	66.7
14	Meng 2018	Y	Y	U	Y	Y	Y	Y	Y	Y	88.9
15	Odinak 2012	Y	NA	NA	N	Y	Y	NA	Y	N	66.7
16	Riordan 2018	Y	NA	NA	N	Y	N	NA	Y	Y	66.7
17	Sahraian 2013	Y	NA	NA	N	Y	Y	NA	Y	N	66.7
18	Sahraian 2019	Y	NA	NA	N	Y	Y	NA	Y	N	66.7
19	Yamout 2010	Y	NA	NA	N	Y	N	NA	Y	N	50.0
20	Cohen 2023	Y	NA	NA	N	Y	Y	NA	Y	Y	55.6
21	Harris 2021	Y	NA	NA	N	Y	Y	NA	Y	Y	55.6
22	Petrou 2021	Y	NA	NA	N	Y	Y	NA	Y	Y	55.6

1. Is it clear in the study what is the 'cause' and what is the 'effect' (i.e. there is no confusion about which variable comes first)? 2. Were the participants included in any comparisons similar? 3. Were the participants included in any comparisons receiving similar treatment/care, other than the exposure or intervention of interest? 4. Was there a control group? 5. Were there multiple measurements of the outcome both pre and post the intervention/exposure? 6. Was follow up complete and if not, were differences between groups in terms of their follow up adequately described and analyzed? 7. Were the outcomes of participants included in any comparisons measured in the same way? 8. Were outcomes measured in a reliable way? 9. Was appropriate statistical analysis used? Abbreviations: Y: Yes, N: No, U: Unclear, NA: Not applicable.

Table S3. Quality assessment of the Randomised controlled trials.

No	Study ID	1	2	3	4	5	6	7	8	9	10	11	12	13	Yes (%)
1	Fernandez 2018	Y	Y	N	Y	Y	Y	Y	N	N	Y	Y	Y	Y	76.9
2	Li 2014	Y	U	Y	Y	U	U	N	U	Y	Y	Y	Y	Y	61.5
3	Lublin 2014	Y	Y	Y	Y	Y	N	U	Y	Y	Y	Y	Y	Y	84.6
4	Nabavi 2023	Y	Y	Y	Y	Y	U	Y	Y	N	Y	Y	Y	Y	84.6
5	Petrou 2020	Y	Y	Y	Y	Y	Y	Y	Y	N	Y	Y	Y	Y	92.3
6	Uccelli 2021	Y	Y	Y	Y	Y	U	Y	Y	N	Y	Y	Y	Y	84.6
7	Tremblay 2022	Y	Y	Y	Y	Y	Y	Y	Y	N	Y	Y	Y	Y	92.3
8	Llufriu 2014	Y	Y	Y	Y	Y	Y	Y	Y	N	Y	Y	Y	Y	92.3

1. Was true randomization used for assignment of participants to treatment groups? 2. Was allocation to treatment groups concealed? 3. Were treatment groups similar at the baseline? 4. Were participants blind to treatment assignment? 5. Were those delivering treatment blind to treatment assignment? 6. Were outcomes assessors blind to treatment assignment? 7. Were treatment groups treated identically other than the intervention of interest? 8. Was follow up complete and if not, were differences between groups in terms of their follow up adequately described and analyzed? 9. Were participants analyzed in the groups to which they were randomized? 10. Were outcomes measured in the same way for treatment groups? 11. Were outcomes measured in a reliable way? 12. Was appropriate statistical analysis used? 13. Was the trial design appropriate, and any deviations from the standard RCT design (individual randomization, parallel groups) accounted for in the conduct and analysis of the trial? Y: Yes, N: No, U: Unclear, NA: Not applicable.

A

Study ID	Cases	Total	Prevalence	95% C.I.	Weight (random)
**Headache**					
Harris 2021	1	18	5.6	[0.0; 16.1]	7.2%
Cohen 2017	2	24	8.3	[0.0; 19.4]	7.2%
Bonab 2012	3	22	13.6	[0.0; 28.0]	7.1%
Iacobaeus 2019	1	5	20.0	[0.0; 55.1]	6.0%
Petrou 2021	11	24	45.8	[25.9; 65.8]	6.8%
Harris 2016	3	6	50.0	[10.0; 90.0]	5.6%
Dahbour 2017	8	15	53.3	[28.1; 78.6]	6.6%
Petrou 2020	19	32	59.4	[42.4; 76.4]	7.0%
Karussis 2010	10	15	66.7	[42.8; 90.5]	6.6%
Riodan 2018	19	25	76.0	[59.3; 92.7]	7.0%
Harris 2018	17	20	85.0	[69.4; 100.0]	7.0%
Cohen 2023	16	18	88.9	[74.4; 100.0]	7.1%
Meng 2018	2	2	100.0	[57.8; 100.0]	5.5%
Sahraian 2013	6	6	100.0	[80.9; 100.0]	6.9%
Sahraian 2019	4	4	100.0	[73.7; 100.0]	6.5%
		**236**			
**Random effects model**			**57.6**	**[37.9; 77.3]**	**100.0%**

Heterogeneity: $I^2 = 94\%$, $\tau^2 = 0.1380$, $\chi^2_{14} = 250.93$ ($p < 0.01$)

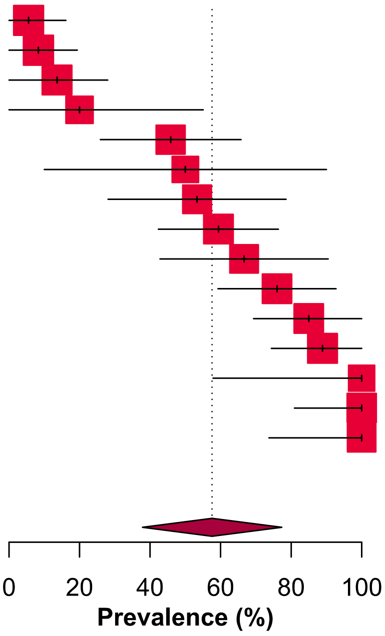

B

Study ID	Cases	Total	Prevalence	95% C.I.	Weight (random)
**Fever**					
Petrou 2020	2	32	6.2	[0.0; 14.6]	10.7%
Harris 2016	1	6	16.7	[0.0; 46.5]	9.9%
Harris 2018	5	20	25.0	[6.0; 44.0]	10.4%
Petrou 2021	6	24	25.0	[7.7; 42.3]	10.4%
Dahbour 2017	6	15	40.0	[15.2; 64.8]	10.1%
Meng 2018	1	2	50.0	[0.0; 100.0]	7.2%
Karussis 2010	10	15	66.7	[42.8; 90.5]	10.2%
Bonab 2012	22	22	100.0	[94.0; 100.0]	10.7%
Shahraian 2013	6	6	100.0	[80.9; 100.0]	10.4%
Sahraian 2019	4	4	100.0	[73.7; 100.0]	10.0%
		**146**			
**Random effects model**			**53.1**	**[20.7; 85.4]**	**100.0%**

Heterogeneity: $I^2 = 98\%$, $\tau^2 = 0.2530$, $\chi^2_{9} = 392.34$ ($p < 0.01$)

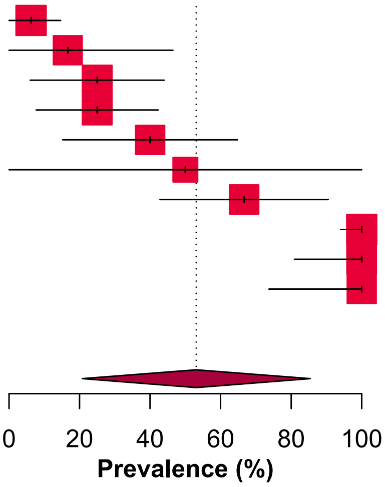

C

Study ID	Cases	Total	Prevalence	95% C.I.	Weight (random)
**Urinary tract infection**					
Petrou 2020	1	32	3.1	[0.0; 9.2]	19.2%
Cornick 2012	1	10	10.0	[0.0; 28.6]	14.9%
Fernandez 2018	3	23	13.0	[0.0; 26.8]	16.8%
Cohen 2023	6	18	33.3	[11.6; 55.1]	13.7%
Iacobaeus 2019	2	5	40.0	[0.0; 82.9]	7.2%
Cohen 2017	10	24	41.7	[21.9; 61.4]	14.5%
Harris 2018	9	20	45.0	[23.2; 66.8]	13.7%
		**132**			
**Random effects model**			**23.9**	**[9.5; 38.3]**	**100.0%**

Heterogeneity: $I^2 = 81\%$, $\tau^2 = 0.0270$, $\chi^2_{6} = 31.07$ ($p < 0.01$)

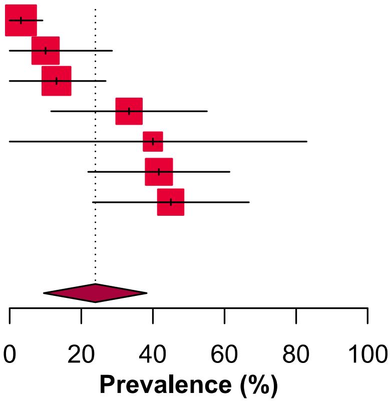

D

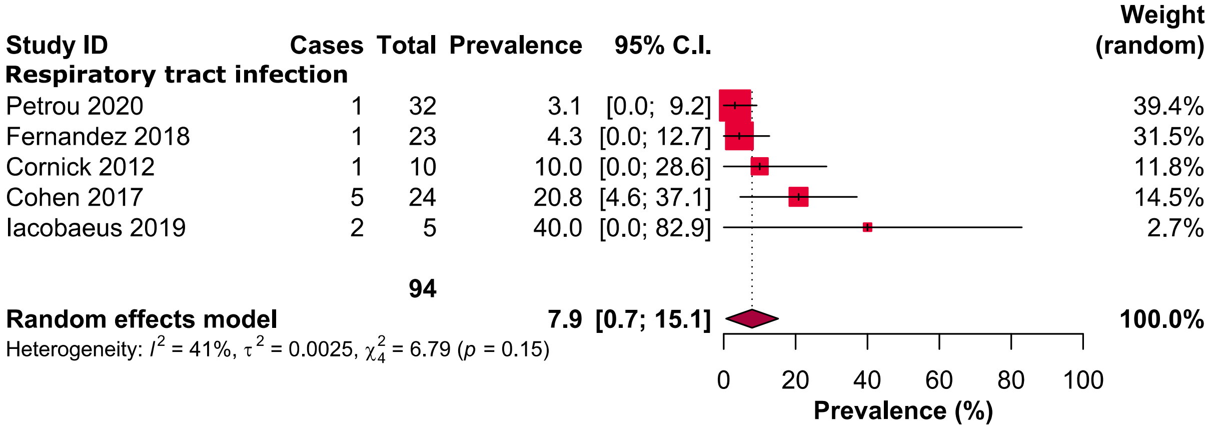
Study ID
Cases
Total
Prevalence
95% C.I.
Weight (random)
Respiratory tract infection
Petrou 2020 1 32 3.1 [0.0; 9.2] 39.4%
Fernandez 2018 1 23 4.3 [0.0; 12.7] 31.5%
Cornick 2012 1 10 10.0 [0.0; 28.6] 11.8%
Cohen 2017 5 24 20.8 [4.6; 37.1] 14.5%
Iacobaeus 2019 2 5 40.0 [0.0; 82.9] 2.7%
94
Random effects model 7.9 [0.7; 15.1] 100.0%
Heterogeneity: I^2 = 41%, τ^2 = 0.0025, χ^2_4 = 6.79 (p = 0.15)
0 20 40 60 80 100
Prevalence (%)

E

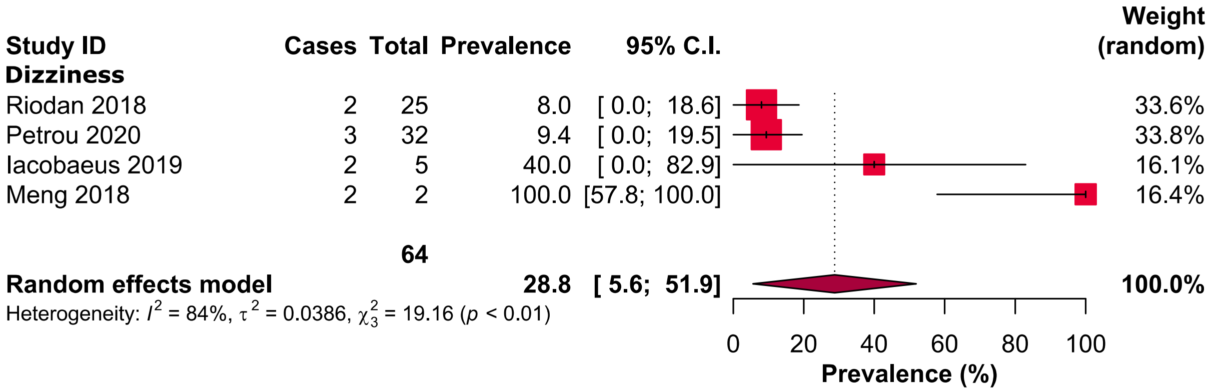
Study ID
Cases
Total
Prevalence
95% C.I.
Weight (random)
Dizziness
Riodan 2018 2 25 8.0 [0.0; 18.6] 33.6%
Petrou 2020 3 32 9.4 [0.0; 19.5] 33.8%
Iacobaeus 2019 2 5 40.0 [0.0; 82.9] 16.1%
Meng 2018 2 2 100.0 [57.8; 100.0] 16.4%
64
Random effects model 28.8 [5.6; 51.9] 100.0%
Heterogeneity: I^2 = 84%, τ^2 = 0.0386, χ^2_3 = 19.16 (p < 0.01)
0 20 40 60 80 100
Prevalence (%)

F

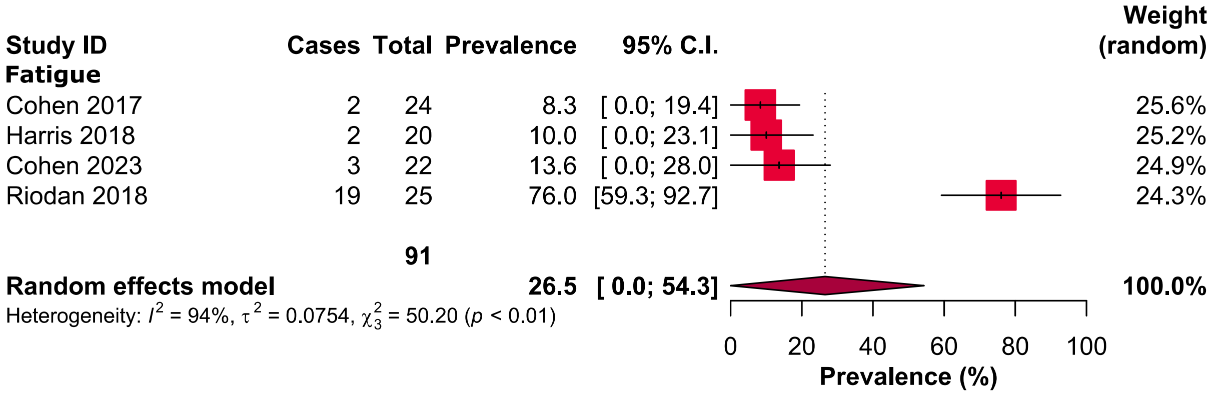
Study ID
Cases
Total
Prevalence
95% C.I.
Weight (random)
Fatigue
Cohen 2017 2 24 8.3 [0.0; 19.4] 25.6%
Harris 2018 2 20 10.0 [0.0; 23.1] 25.2%
Cohen 2023 3 22 13.6 [0.0; 28.0] 24.9%
Riodan 2018 19 25 76.0 [59.3; 92.7] 24.3%
91
Random effects model 26.5 [0.0; 54.3] 100.0%
Heterogeneity: I^2 = 94%, τ^2 = 0.0754, χ^2_3 = 50.20 (p < 0.01)
0 20 40 60 80 100
Prevalence (%)

G

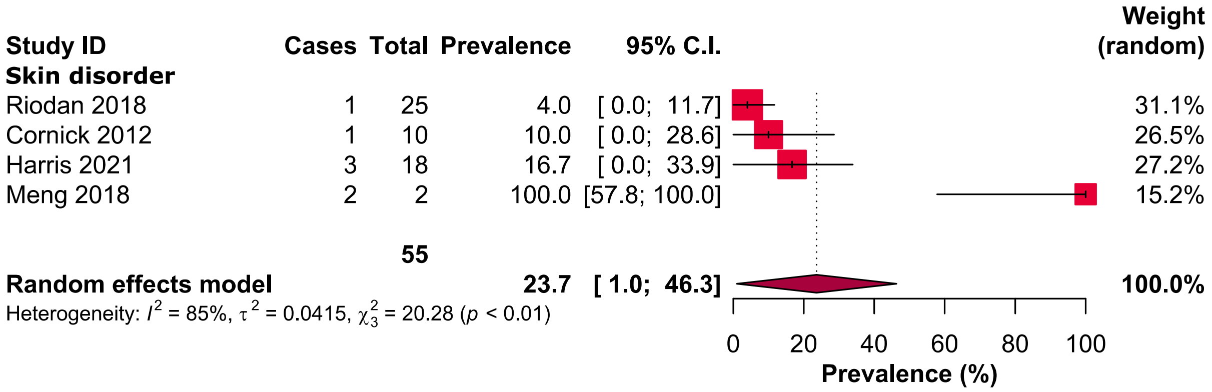
Study ID
Cases
Total
Prevalence
95% C.I.
Weight (random)
Skin disorder
Riodan 2018 1 25 4.0 [0.0; 11.7] 31.1%
Cornick 2012 1 10 10.0 [0.0; 28.6] 26.5%
Harris 2021 3 18 16.7 [0.0; 33.9] 27.2%
Meng 2018 2 2 100.0 [57.8; 100.0] 15.2%
55
Random effects model 23.7 [1.0; 46.3] 100.0%
Heterogeneity: I^2 = 85%, τ^2 = 0.0415, χ^2_3 = 20.28 (p < 0.01)
0 20 40 60 80 100
Prevalence (%)

H

Study ID	Cases	Total	Prevalence	95% C.I.	Weight (random)
**Back pain**					
Cohen 2017	2	24	8.3	[0.0; 19.4]	21.2%
Petrou 2021	3	24	12.5	[0.0; 25.7]	20.8%
Petrou 2020	4	32	12.5	[1.0; 24.0]	21.1%
Yamout 2010	1	6	16.7	[0.0; 46.5]	16.8%
Cohen 2023	15	18	83.3	[66.1; 100.0]	20.0%
		**104**			
**Random effects model**			**26.5**	**[1.5; 51.5]**	**100.0%**

Heterogeneity: $I^2 = 93\%$, $\tau^2 = 0.0738$, $\chi^2_4 = 59.16$ ($p < 0.01$)

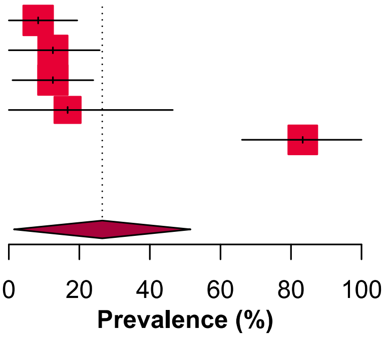

I

Study ID	Cases	Total	Prevalence	95% C.I.	Weight (random)
**Balance disorder**					
Cohen 2017	5	24	20.8	[4.6; 37.1]	65.5%
Karussis 2010	4	15	26.7	[4.3; 49.0]	34.5%
		**39**			
**Random effects model**			**22.8**	**[9.7; 36.0]**	**100.0%**

Heterogeneity: $I^2 = 0\%$, $\tau^2 = 0$, $\chi^2_1 = 0.17$ ($p = 0.68$)

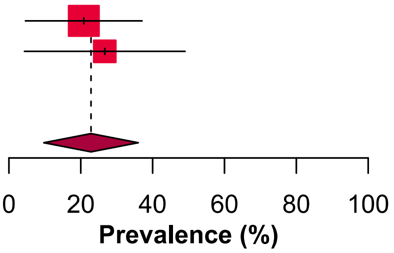

J

Study ID	Cases	Total	Prevalence	95% C.I.	Weight (random)
**Depression**					
Harris 2018	1	20	5.0	[0.0; 14.6]	65.7%
Cohen 2017	3	24	12.5	[0.0; 25.7]	34.3%
		**44**			
**Random effects model**			**7.6**	**[0.0; 15.3]**	**100.0%**

Heterogeneity: $I^2 = 0\%$, $\tau^2 = 0$, $\chi^2_1 = 0.81$ ($p = 0.37$)

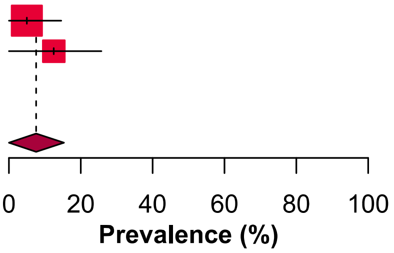

K

Study ID	Cases	Total	Prevalence	95% C.I.	Weight (random)
**Fall**					
Petrou 2020	3	32	9.4	[0.0; 19.5]	43.4%
Cohen 2023	3	18	16.7	[0.0; 33.9]	26.0%
Cohen 2017	7	24	29.2	[11.0; 47.4]	24.3%
Iacobaeus 2019	2	5	40.0	[0.0; 82.9]	6.2%
		**79**			
**Random effects model**			**18.0**	**[6.7; 29.2]**	**100.0%**

Heterogeneity: $I^2 = 38\%$, $\tau^2 = 0.0049$, $\chi^2_3 = 4.85$ ($p = 0.18$)

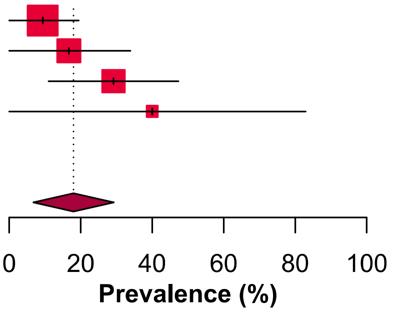

L

Study ID	Cases	Total	Prevalence	95% C.I.	Weight (random)
**Rash**					
Petrou 2020	1	32	3.1	[0.0; 9.2]	88.1%
Cornick 2012	1	10	10.0	[0.0; 28.6]	9.3%
Iacobaeus 2019	1	5	20.0	[0.0; 55.1]	2.6%
		**47**			
**Random effects model**			**4.2**	**[0.0; 9.9]**	**100.0%**

Heterogeneity: $I^2 = 0\%$, $\tau^2 = 0$, $\chi^2_2 = 1.28$ ($p = 0.53$)

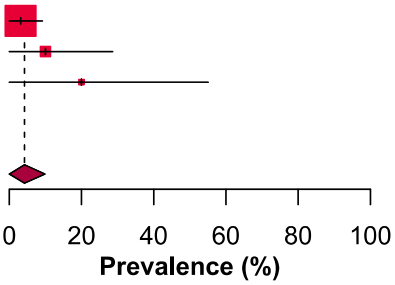

M

Study ID	Cases	Total	Prevalence	95% C.I.	Weight (random)
**Musculoskeletal stiffness**					
Harris 2021	2	18	11.1	[0.0; 25.6]	36.5%
Karussis 2010	2	15	13.3	[0.0; 30.5]	26.0%
Cohen 2023	3	22	13.6	[0.0; 28.0]	37.4%
		**55**			
**Random effects model**			**12.6**	**[3.9; 21.4]**	**100.0%**

Heterogeneity: $I^2 = 0\%$, $\tau^2 = 0$, $\chi^2_2 = 0.07$ ($p = 0.97$)

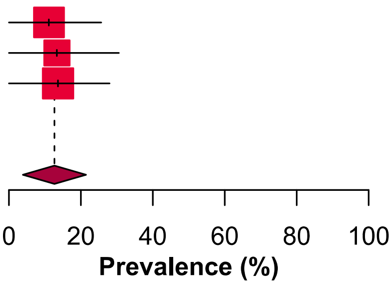

N

Study ID	Cases	Total	Prevalence	95% C.I.	Weight (random)
**Sinusitis**					
Petrou 2020	2	32	6.2	[0.0; 14.6]	70.3%
Iacobaeus 2019	2	5	40.0	[0.0; 82.9]	29.7%
		**37**			
**Random effects model**			**16.3**	**[0.0; 46.5]**	**100.0%**

Heterogeneity: $I^2 = 56\%$, $\tau^2 = 0.0320$, $\chi^2_1 = 2.29$ ($p = 0.13$)

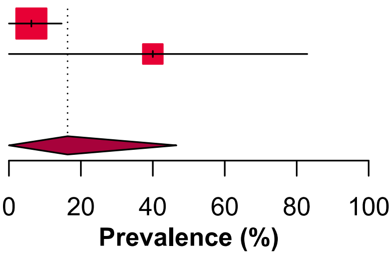

O

Study ID	Cases	Total	Prevalence	95% C.I.	Weight (random)
**Cervical pain**					
Petrou 2020	1	32	3.1	[0.0; 9.2]	96.1%
Yamout 2010	1	6	16.7	[0.0; 46.5]	3.9%
		**38**			
**Random effects model**			**3.7**	**[0.0; 9.6]**	**100.0%**

Heterogeneity: $I^2 = 0\%$, $\tau^2 = 0$, $\chi^2_1 = 0.76$ ($p = 0.38$)

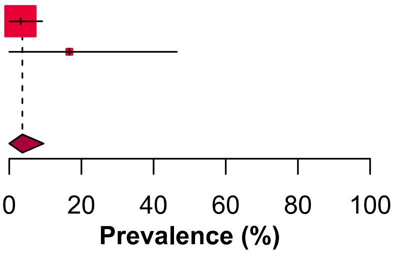

P

Study ID	Cases	Total	Prevalence	95% C.I.	Weight (random)
**Injection site pain**					
Cohen 2023	4	18	22.2	[3.0; 41.4]	52.2%
Dahbour 2017	9	15	60.0	[35.2; 84.8]	47.8%
		**33**			
**Random effects model**			**40.3**	**[3.3; 77.2]**	**100.0%**

Heterogeneity: $I^2 = 82\%$, $\tau^2 = 0.0586$, $\chi^2_1 = 5.57$ ($p = 0.02$)

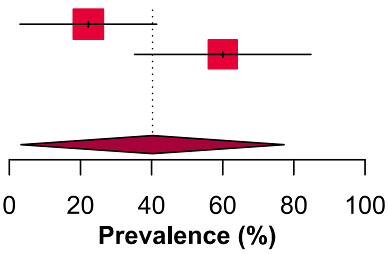

Figure S1. Adverse events in patients with multiple sclerosis following the mesenchymal stem cell therapy. A) headache, B) fever, C) urinary tract infection, D) respiratory tract infection, E) dizziness, F) fatigue, G) skin disorder, H) back pain, I) balance disorder, J) depression, K) fall, L) rash, M) Musculoskeletal stiffness, N) sinusitis, O) cervical pain, and P) injection site pain.

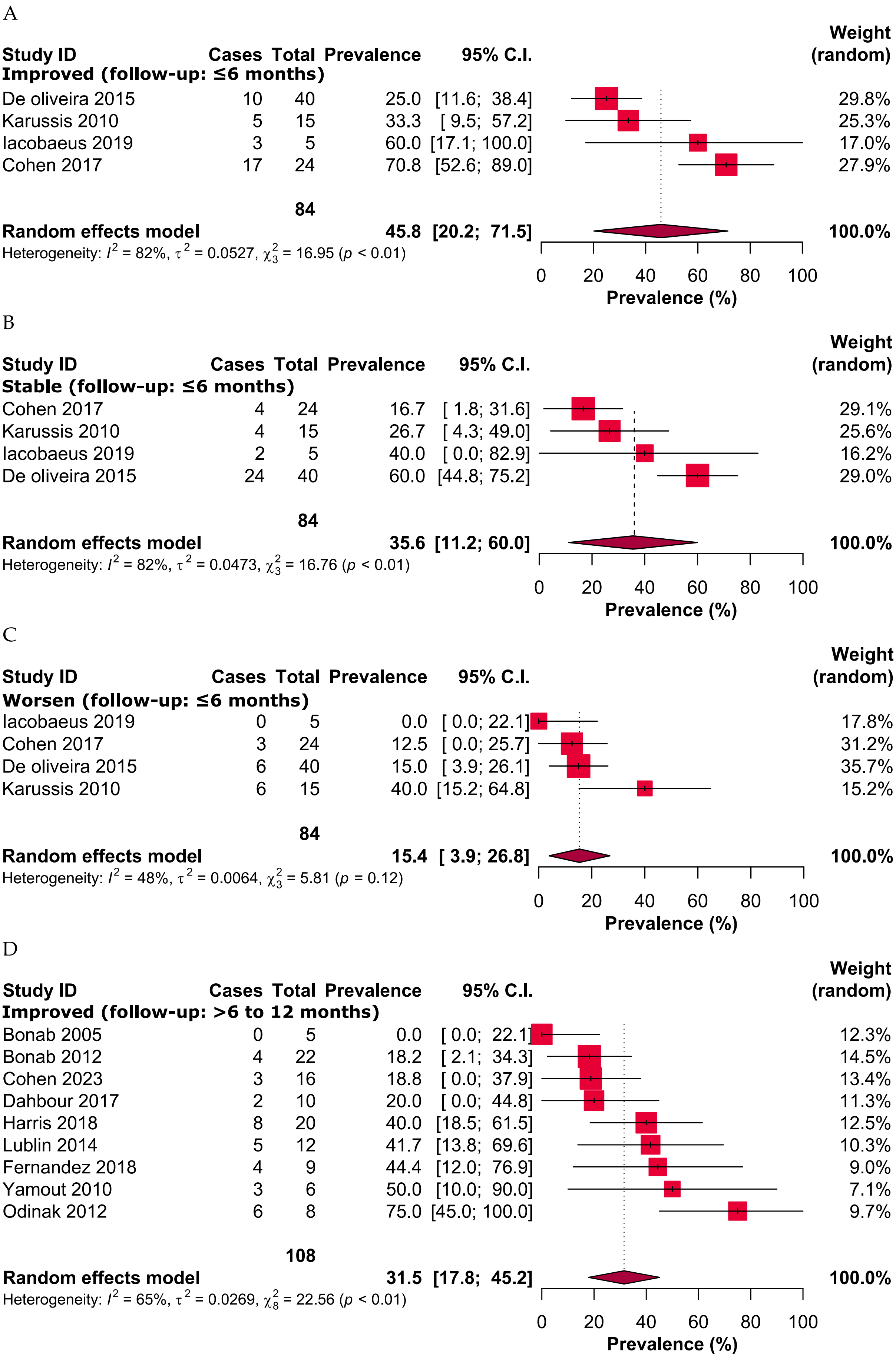
A
Study ID Cases Total Prevalence 95% C.I. Weight (random)
Improved (follow-up: ≤6 months)
De oliveira 2015 10 40 25.0 [11.6; 38.4] 29.8%
Karussis 2010 5 15 33.3 [9.5; 57.2] 25.3%
Iacobaeus 2019 3 5 60.0 [17.1; 100.0] 17.0%
Cohen 2017 17 24 70.8 [52.6; 89.0] 27.9%
84
Random effects model 45.8 [20.2; 71.5] 100.0%
Heterogeneity: I^2 = 82%, τ^2 = 0.0527, χ^2_3 = 16.95 (p < 0.01)
0 20 40 60 80 100
Prevalence (%)
B
Study ID Cases Total Prevalence 95% C.I. Weight (random)
Stable (follow-up: ≤6 months)
Cohen 2017 4 24 16.7 [1.8; 31.6] 29.1%
Karussis 2010 4 15 26.7 [4.3; 49.0] 25.6%
Iacobaeus 2019 2 5 40.0 [0.0; 82.9] 16.2%
De oliveira 2015 24 40 60.0 [44.8; 75.2] 29.0%
84
Random effects model 35.6 [11.2; 60.0] 100.0%
Heterogeneity: I^2 = 82%, τ^2 = 0.0473, χ^2_3 = 16.76 (p < 0.01)
0 20 40 60 80 100
Prevalence (%)
C
Study ID Cases Total Prevalence 95% C.I. Weight (random)
Worsen (follow-up: ≤6 months)
Iacobaeus 2019 0 5 0.0 [0.0; 22.1] 17.8%
Cohen 2017 3 24 12.5 [0.0; 25.7] 31.2%
De oliveira 2015 6 40 15.0 [3.9; 26.1] 35.7%
Karussis 2010 6 15 40.0 [15.2; 64.8] 15.2%
84
Random effects model 15.4 [3.9; 26.8] 100.0%
Heterogeneity: I^2 = 48%, τ^2 = 0.0064, χ^2_3 = 5.81 (p = 0.12)
0 20 40 60 80 100
Prevalence (%)
D
Study ID Cases Total Prevalence 95% C.I. Weight (random)
Improved (follow-up: >6 to 12 months)
Bonab 2005 0 5 0.0 [0.0; 22.1] 12.3%
Bonab 2012 4 22 18.2 [2.1; 34.3] 14.5%
Cohen 2023 3 16 18.8 [0.0; 37.9] 13.4%
Dahbour 2017 2 10 20.0 [0.0; 44.8] 11.3%
Harris 2018 8 20 40.0 [18.5; 61.5] 12.5%
Lublin 2014 5 12 41.7 [13.8; 69.6] 10.3%
Fernandez 2018 4 9 44.4 [12.0; 76.9] 9.0%
Yamout 2010 3 6 50.0 [10.0; 90.0] 7.1%
Odinak 2012 6 8 75.0 [45.0; 100.0] 9.7%
108
Random effects model 31.5 [17.8; 45.2] 100.0%
Heterogeneity: I^2 = 65%, τ^2 = 0.0269, χ^2_8 = 22.56 (p < 0.01)
0 20 40 60 80 100
Prevalence (%)

E

Study ID	Cases	Total	Prevalence	95% C.I.	Weight (random)
**Stable (follow-up: >6 to 12 months)**					
Odinak 2012	1	8	12.5	[0.0; 35.4]	14.7%
Yamout 2010	1	6	16.7	[0.0; 46.5]	11.1%
Fernandez 2018	2	9	22.2	[0.0; 49.4]	12.4%
Lublin 2014	4	12	33.3	[6.7; 60.0]	12.6%
Bonab 2005	2	5	40.0	[0.0; 82.9]	6.8%
Dahbour 2017	4	10	40.0	[9.6; 70.4]	10.9%
Harris 2018	10	20	50.0	[28.1; 71.9]	15.3%
Bonab 2012	13	22	59.1	[38.5; 79.6]	16.2%
		**92**			
**Random effects model**			**34.9**	**[22.0; 47.9]**	**100.0%**

Heterogeneity: $I^2 = 47\%$, $\tau^2 = 0.0159$, $\chi^2_7 = 13.20$ ($p = 0.07$)

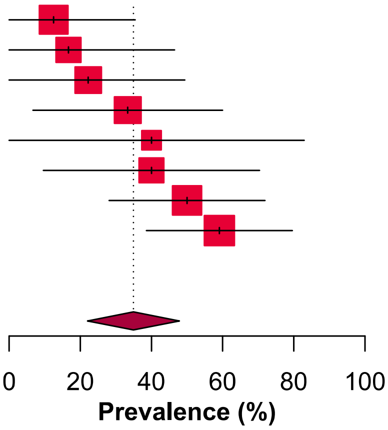

F

Study ID	Cases	Total	Prevalence	95% C.I.	Weight (random)
**Worsen (follow-up: >6 to 12 months)**					
Harris 2018	2	20	10.0	[0.0; 23.1]	27.5%
Odinak 2012	1	8	12.5	[0.0; 35.4]	13.4%
Bonab 2012	5	22	22.7	[5.2; 40.2]	19.7%
Lublin 2014	3	12	25.0	[0.5; 49.5]	12.1%
Fernandez 2018	3	9	33.3	[2.5; 64.1]	8.3%
Yamout 2010	2	6	33.3	[0.0; 71.1]	5.8%
Dahbour 2017	4	10	40.0	[9.6; 70.4]	8.5%
Bonab 2005	3	5	60.0	[17.1; 100.0]	4.6%
		**92**			
**Random effects model**			**22.8**	**[13.2; 32.4]**	**100.0%**

Heterogeneity: $I^2 = 23\%$, $\tau^2 = 0.0042$, $\chi^2_7 = 9.05$ ($p = 0.25$)

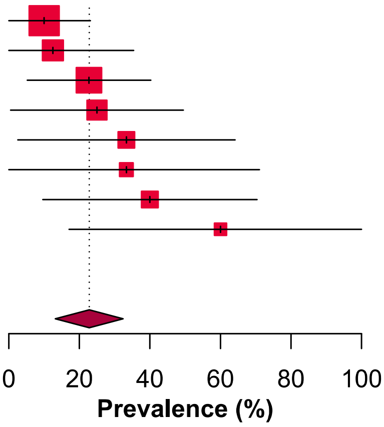

G

Study ID	Cases	Total	Prevalence	95% C.I.	Weight (random)
**Improved (follow-up: >12 months)**					
Bonab 2007	1	10	10.0	[0.0; 28.6]	15.6%
Sahraian 2013	2	6	33.3	[0.0; 71.1]	9.7%
Harris 2021	7	18	38.9	[16.4; 61.4]	14.3%
Meng 2018	1	2	50.0	[0.0; 100.0]	4.5%
Sahraian 2019	2	4	50.0	[1.0; 99.0]	7.3%
Petrou 2020	17	32	53.1	[35.8; 70.4]	16.0%
Harris 2016	4	6	66.7	[28.9; 100.0]	9.7%
Petrou 2021	5	7	71.4	[38.0; 100.0]	10.9%
Lu 2013	6	8	75.0	[45.0; 100.0]	11.9%
		**93**			
**Random effects model**			**48.0**	**[31.3; 64.7]**	**100.0%**

Heterogeneity: $I^2 = 65\%$, $\tau^2 = 0.0377$, $\chi^2_8 = 22.56$ ($p < 0.01$)

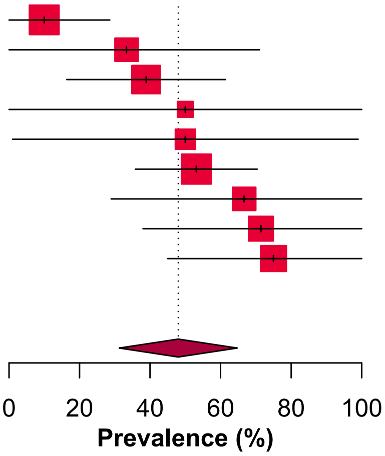

H

Study ID	Cases	Total	Prevalence	95% C.I.	Weight (random)
**Stable (follow-up: >12 months)**					
Lu 2013	1	8	12.5	[0.0; 35.4]	15.7%
Sahraian 2013	1	6	16.7	[0.0; 46.5]	9.3%
Sahraian 2019	1	4	25.0	[0.0; 67.4]	4.6%
Petrou 2021	2	7	28.6	[0.0; 62.0]	7.4%
Harris 2016	2	6	33.3	[0.0; 71.1]	5.8%
Harris 2021	6	18	33.3	[11.6; 55.1]	17.4%
Petrou 2020	12	32	37.5	[20.7; 54.3]	29.3%
Bonab 2007	4	10	40.0	[9.6; 70.4]	8.9%
Meng 2018	1	2	50.0	[0.0; 100.0]	1.7%
		**93**			
**Random effects model**			**29.9**	**[20.8; 39.0]**	**100.0%**

Heterogeneity: $I^2 = 0\%$, $\tau^2 = 0$, $\chi^2_8 = 4.69$ ($p = 0.79$)

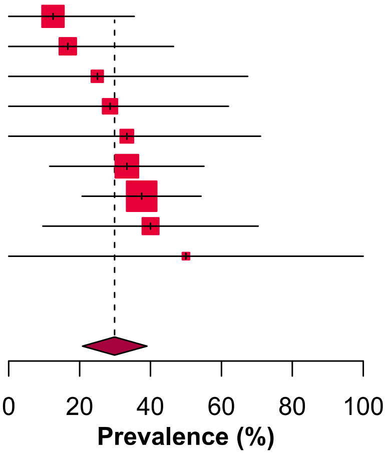

I

Study ID	Cases	Total	Prevalence	95% C.I.	Weight (random)
**Worsen (follow-up: >12 months)**					
Harris 2016	0	6	0.0	[0.0; 19.1]	14.2%
Meng 2018	0	2	0.0	[0.0; 42.2]	5.2%
Petrou 2021	0	7	0.0	[0.0; 16.8]	15.8%
Petrou 2020	3	32	9.4	[0.0; 19.5]	20.9%
Lu 2013	1	8	12.5	[0.0; 35.4]	11.8%
Sahraian 2019	1	4	25.0	[0.0; 67.4]	5.1%
Harris 2021	5	18	27.8	[7.1; 48.5]	13.1%
Bonab 2007	5	10	50.0	[19.0; 81.0]	8.2%
Sahraian 2013	3	6	50.0	[10.0; 90.0]	5.6%
		**93**			
**Random effects model**			**15.3**	**[4.5; 26.0]**	**100.0%**

Heterogeneity: $I^2 = 50\%$, $\tau^2 = 0.0117$, $\chi^2_8 = 15.89$ ($p = 0.04$)

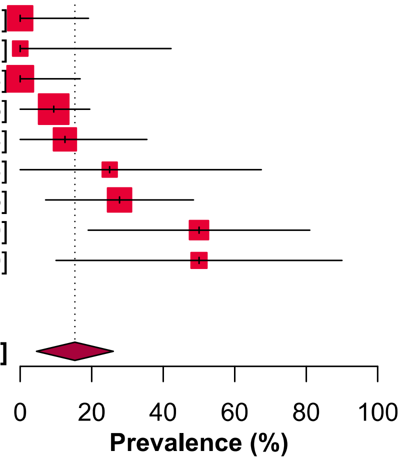

J

Study ID	Cases	Total	Prevalence	95% C.I.	Weight (random)
**Improved (bone marrow-derived stem cells)**					
Bonab 2005	0	5	0.0	[0.0; 22.1]	5.9%
Bonab 2007	1	10	10.0	[0.0; 28.6]	6.4%
Bonab 2012	4	22	18.2	[2.1; 34.3]	6.8%
Cohen 2023	3	16	18.8	[0.0; 37.9]	6.3%
Dahbour 2017	2	10	20.0	[0.0; 44.8]	5.5%
De oliveira 2015	10	40	25.0	[11.6; 38.4]	7.1%
Karussis 2010	5	15	33.3	[9.5; 57.2]	5.7%
Sahraian 2013	2	6	33.3	[0.0; 71.1]	3.9%
Harris 2021	7	18	38.9	[16.4; 61.4]	5.8%
Harris 2018	8	20	40.0	[18.5; 61.5]	6.0%
Fernandez 2018	4	9	44.4	[12.0; 76.9]	4.5%
Sahraian 2019	2	4	50.0	[1.0; 99.0]	2.9%
Yamout 2010	3	6	50.0	[10.0; 90.0]	3.7%
Petrou 2020	17	32	53.1	[35.8; 70.4]	6.6%
Iacobaeus 2019	3	5	60.0	[17.1; 100.0]	3.4%
Harris 2016	4	6	66.7	[28.9; 100.0]	3.9%
Cohen 2017	17	24	70.8	[52.6; 89.0]	6.5%
Petrou 2021	5	7	71.4	[38.0; 100.0]	4.4%
Odinak 2012	6	8	75.0	[45.0; 100.0]	4.8%
		**263**			
**Random effects model**			**38.5**	**[28.2; 48.9]**	**100.0%**

Heterogeneity: $I^2 = 71\%$, $\tau^2 = 0.0345$, $\chi^2_{18} = 63.15$ ($p < 0.01$)

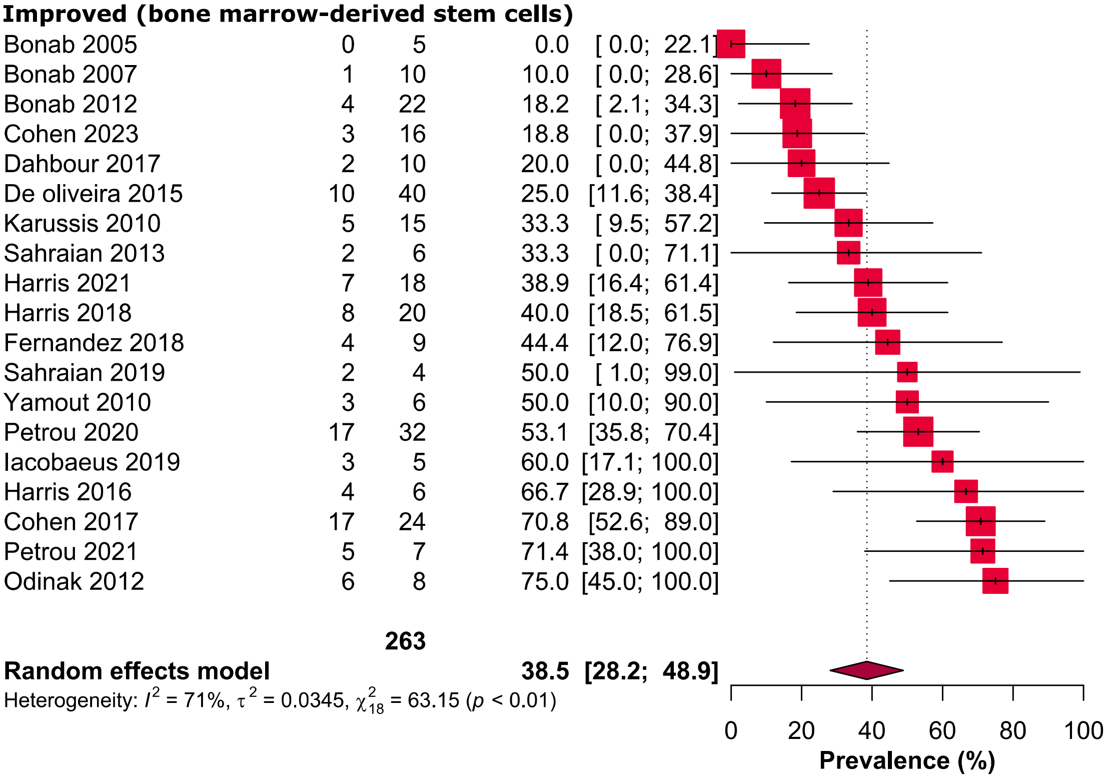

K

Study ID	Cases	Total	Prevalence	95% C.I.	Weight (random)
**Stable (bone marrow-derived stem cells)**					
Odinak 2012	1	8	12.5	[0.0; 35.4]	6.4%
Cohen 2017	4	24	16.7	[1.8; 31.6]	9.0%
Sahraian 2013	1	6	16.7	[0.0; 46.5]	4.7%
Yamout 2010	1	6	16.7	[0.0; 46.5]	4.7%
Fernandez 2018	2	9	22.2	[0.0; 49.4]	5.3%
Sahraian 2019	1	4	25.0	[0.0; 67.4]	2.9%
Karussis 2010	4	15	26.7	[4.3; 49.0]	6.6%
Petrou 2021	2	7	28.6	[0.0; 62.0]	4.1%
Harris 2016	2	6	33.3	[0.0; 71.1]	3.4%
Harris 2021	6	18	33.3	[11.6; 55.1]	6.7%
Petrou 2020	12	32	37.5	[20.7; 54.3]	8.4%
Bonab 2005	2	5	40.0	[0.0; 82.9]	2.8%
Bonab 2007	4	10	40.0	[9.6; 70.4]	4.6%
Dahbour 2017	4	10	40.0	[9.6; 70.4]	4.6%
Iacobaeus 2019	2	5	40.0	[0.0; 82.9]	2.8%
Harris 2018	10	20	50.0	[28.1; 71.9]	6.7%
Bonab 2012	13	22	59.1	[38.5; 79.6]	7.1%
De oliveira 2015	24	40	60.0	[44.8; 75.2]	8.9%
		**247**			
**Random effects model**			**34.1**	**[26.0; 42.3]**	**100.0%**

Heterogeneity: $I^2 = 47\%$, $\tau^2 = 0.0134$, $\chi^2_{17} = 32.08$ ($p = 0.01$)

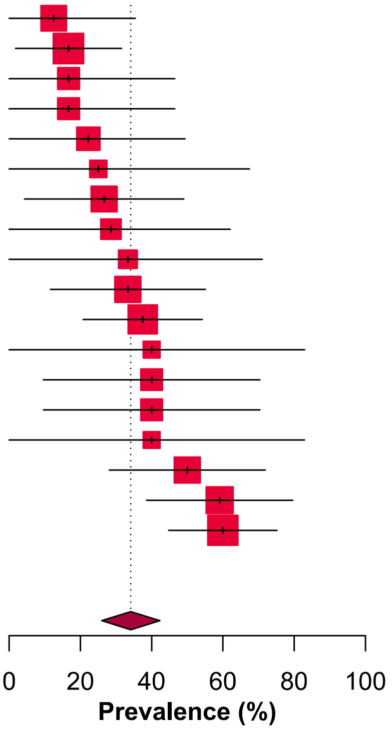

L

Study ID	Cases	Total	Prevalence	95% C.I.	Weight (random)
**Worsen (bone marrow-derived stem cells)**					
Harris 2016	0	6	0.0	[0.0; 19.1]	6.5%
Iacobaeus 2019	0	5	0.0	[0.0; 22.1]	5.5%
Petrou 2021	0	7	0.0	[0.0; 16.8]	7.3%
Petrou 2020	3	32	9.4	[0.0; 19.5]	10.4%
Harris 2018	2	20	10.0	[0.0; 23.1]	8.9%
Cohen 2017	3	24	12.5	[0.0; 25.7]	8.9%
Odinak 2012	1	8	12.5	[0.0; 35.4]	5.2%
De oliveira 2015	6	40	15.0	[3.9; 26.1]	9.9%
Bonab 2012	5	22	22.7	[5.2; 40.2]	7.1%
Sahraian 2019	1	4	25.0	[0.0; 67.4]	2.1%
Harris 2021	5	18	27.8	[7.1; 48.5]	5.9%
Fernandez 2018	3	9	33.3	[2.5; 64.1]	3.5%
Yamout 2010	2	6	33.3	[0.0; 71.1]	2.6%
Dahbour 2017	4	10	40.0	[9.6; 70.4]	3.6%
Karussis 2010	6	15	40.0	[15.2; 64.8]	4.7%
Bonab 2007	5	10	50.0	[19.0; 81.0]	3.5%
Sahraian 2013	3	6	50.0	[10.0; 90.0]	2.3%
Bonab 2005	3	5	60.0	[17.1; 100.0]	2.1%
		**247**			
**Random effects model**			**18.4**	**[11.7; 25.0]**	**100.0%**

Heterogeneity: $I^2 = 48\%$, $\tau^2 = 0.0085$, $\chi^2_{17} = 32.39$ ($p = 0.01$)

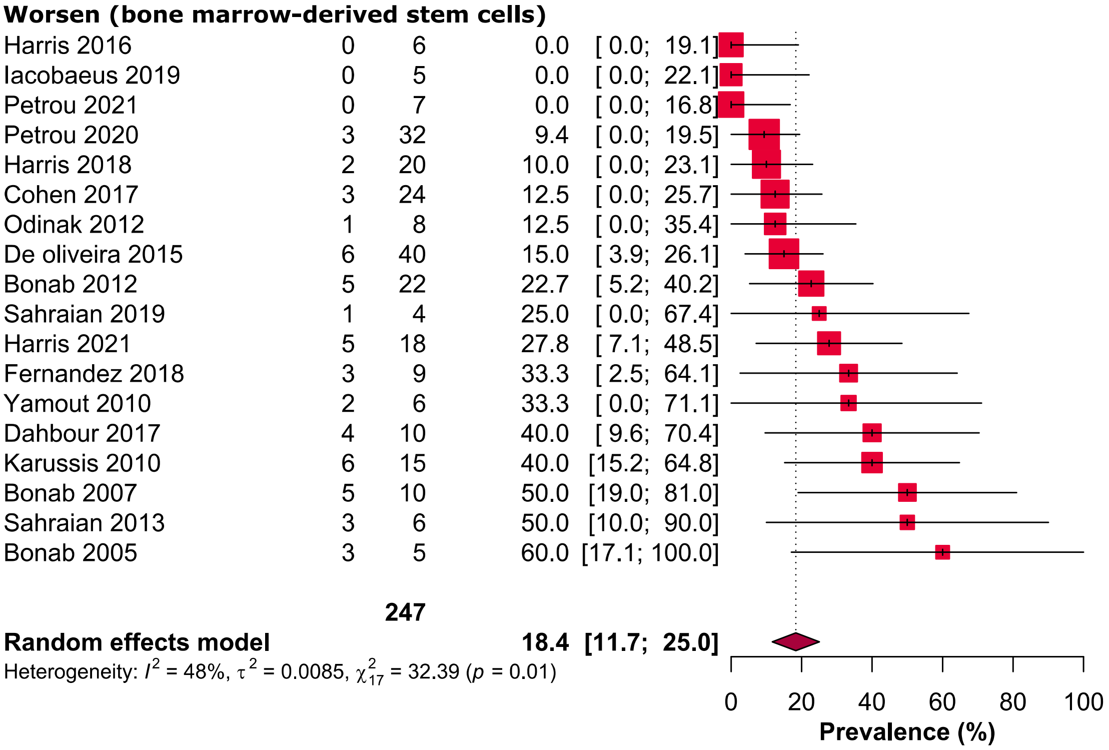

M

Study ID	Cases	Total	Prevalence	95% C.I.	Weight (random)
**Improved (umbilical cord or placenta-derived stem cells)**					
Lublin 2014	5	12	41.7	[13.8; 69.6]	46.9%
Meng 2018	1	2	50.0	[0.0; 100.0]	10.6%
Lu 2013	6	8	75.0	[45.0; 100.0]	42.5%
		**22**			
**Random effects model**			**56.7**	**[33.3; 80.1]**	**100.0%**

Heterogeneity: $I^2 = 22\%$, $\tau^2 = 0.0102$, $\chi^2_{2} = 2.58$ ($p = 0.28$)

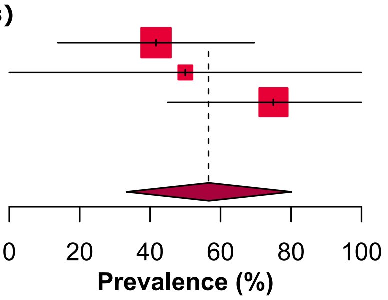

N

Study ID	Cases	Total	Prevalence	95% C.I.	Weight (random)
**Stable (umbilical cord or placenta-derived stem cells)**					
Lu 2013	1	8	12.5	[0.0; 35.4]	54.1%
Lublin 2014	4	12	33.3	[6.7; 60.0]	40.0%
Meng 2018	1	2	50.0	[0.0; 100.0]	5.9%
		**22**			**--**
**Random effects model**			**23.0**	**[6.2; 39.9]**	**100.0%**

Heterogeneity: $I^2 = 0\%$, $\tau^2 = 0$, $\chi^2_{2} = 1.97$ ($p = 0.37$)

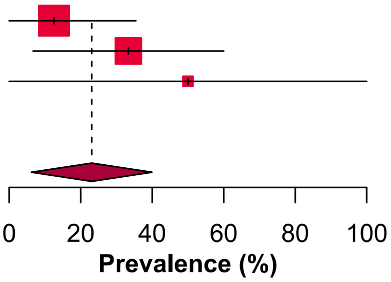

O

Study ID	Cases	Total	Prevalence	95% C.I.	Weight (random)
**Worsen (umbilical cord or placenta-derived stem cells)**					
Meng 2018	0	2	0.0	[0.0; 42.2]	13.6%
Lu 2013	1	8	12.5	[0.0; 35.4]	46.1%
Lublin 2014	3	12	25.0	[0.5; 49.5]	40.3%
		**22**			
**Random effects model**			**15.8**	**[0.3; 31.4]**	**100.0%**

Heterogeneity: I^2 = 0%, τ^2 = 0, χ^2_2 = 1.16 (p = 0.56)

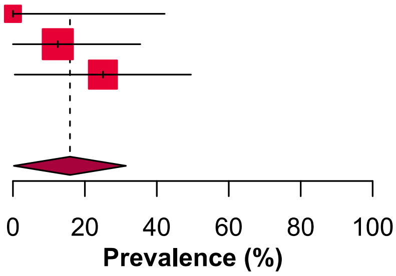

P

Study ID	Cases	Total	Prevalence	95% C.I.	Weight (random)
**Improved (Intravenous)**					
Petrou 2020	6	16	37.5	[13.8; 61.2]	18.3%
Lublin 2014	5	12	41.7	[13.8; 69.6]	15.0%
Fernandez 2018	4	9	44.4	[12.0; 76.9]	12.2%
Meng 2018	1	2	50.0	[0.0; 100.0]	3.5%
Cohen 2017	17	24	70.8	[52.6; 89.0]	23.8%
Lu 2013	6	8	75.0	[45.0; 100.0]	13.6%
Odinak 2012	6	8	75.0	[45.0; 100.0]	13.6%
		**79**			
**Random effects model**			**57.6**	**[44.1; 71.0]**	**100.0%**

Heterogeneity: I^2 = 35%, τ^2 = 0.0112, χ^2_6 = 9.28 (p = 0.16)

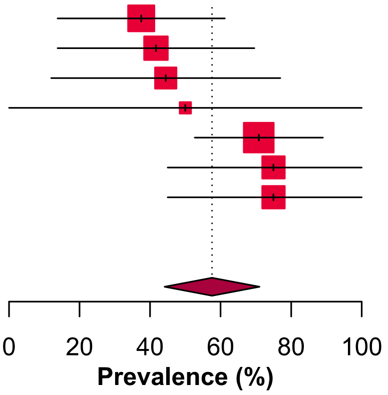

Q

Study ID	Cases	Total	Prevalence	95% C.I.	Weight (random)
**Stable (Intravenous)**					
Lu 2013	1	8	12.5	[0.0; 35.4]	16.9%
Odinak 2012	1	8	12.5	[0.0; 35.4]	16.9%
Cohen 2017	4	24	16.7	[1.8; 31.6]	39.9%
Fernandez 2018	2	9	22.2	[0.0; 49.4]	12.0%
Lublin 2014	4	12	33.3	[6.7; 60.0]	12.5%
Meng 2018	1	2	50.0	[0.0; 100.0]	1.8%
		**63**			
**Random effects model**			**18.6**	**[9.2; 28.0]**	**100.0%**

Heterogeneity: I^2 = 0%, τ^2 = 0, χ^2_5 = 2.64 (p = 0.76)

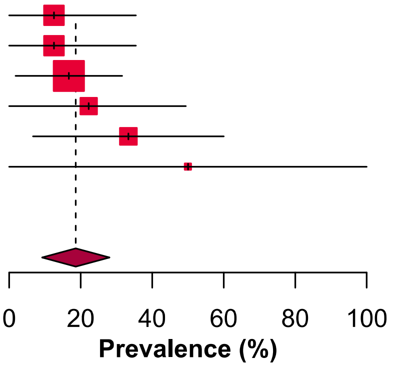

R

Study ID	Cases	Total	Prevalence	95% C.I.	Weight (random)
**Worsen (Intravenous)**					
Meng 2018	0	2	0.0	[0.0; 42.2]	3.6%
Cohen 2017	3	24	12.5	[0.0; 25.7]	36.8%
Lu 2013	1	8	12.5	[0.0; 35.4]	12.3%
Odinak 2012	1	8	12.5	[0.0; 35.4]	12.3%
Petrou 2020	3	16	18.8	[0.0; 37.9]	17.6%
Lublin 2014	3	12	25.0	[0.5; 49.5]	10.7%
Fernandez 2018	3	9	33.3	[2.5; 64.1]	6.8%
		**79**			
**Random effects model**			**15.9**	**[7.9; 23.9]**	**100.0%**

Heterogeneity: I^2 = 0%, τ^2 = 0, χ^2_6 = 2.82 (p = 0.83)

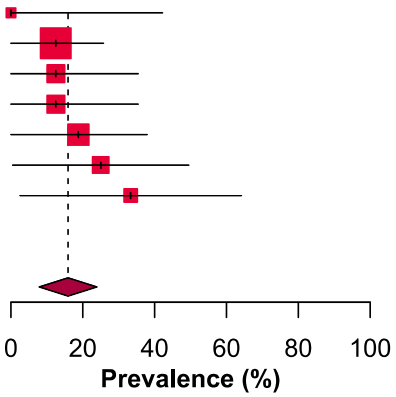

S

Study ID	Cases	Total	Prevalence	95% C.I.	Weight (random)
**Improved (Intrathecal)**					
Bonab 2005	0	5	0.0	[0.0; 22.1]	8.3%
Bonab 2007	1	10	10.0	[0.0; 28.6]	9.2%
Bonab 2012	4	22	18.2	[2.1; 34.3]	9.8%
Cohen 2023	3	16	18.8	[0.0; 37.9]	9.1%
Dahbour 2017	2	10	20.0	[0.0; 44.8]	7.7%
Karussis 2010	5	15	33.3	[9.5; 57.2]	7.9%
Sahraian 2013	2	6	33.3	[0.0; 71.1]	5.1%
Harris 2021	7	18	38.9	[16.4; 61.4]	8.2%
Harris 2018	8	20	40.0	[18.5; 61.5]	8.5%
Sahraian 2019	2	4	50.0	[1.0; 99.0]	3.7%
Yamout 2010	3	6	50.0	[10.0; 90.0]	4.8%
Iacobaeus 2019	3	5	60.0	[17.1; 100.0]	4.4%
Harris 2016	4	6	66.7	[28.9; 100.0]	5.1%
Petrou 2020	11	16	68.8	[46.0; 91.5]	8.2%
		**159**			
**Random effects model**			**32.8**	**[21.6; 44.0]**	**100.0%**

Heterogeneity: I^2 = 63%, τ^2 = 0.0267, χ^2_{13} = 35.25 (p < 0.01)

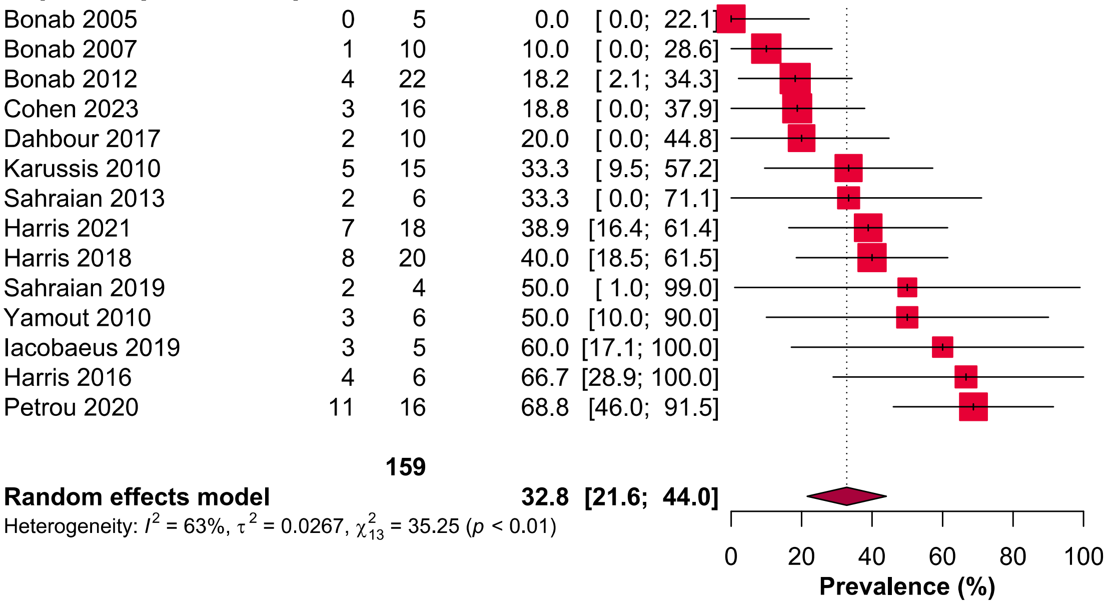

T

Study ID	Cases	Total	Prevalence	95% C.I.	Weight (random)
**Stable (Intrathecal)**					
Sahraian 2013	1	6	16.7	[0.0; 46.5]	7.3%
Yamout 2010	1	6	16.7	[0.0; 46.5]	7.3%
Sahraian 2019	1	4	25.0	[0.0; 67.4]	3.6%
Karussis 2010	4	15	26.7	[4.3; 49.0]	13.0%
Harris 2016	2	6	33.3	[0.0; 71.1]	4.6%
Harris 2021	6	18	33.3	[11.6; 55.1]	13.8%
Bonab 2005	2	5	40.0	[0.0; 82.9]	3.5%
Bonab 2007	4	10	40.0	[9.6; 70.4]	7.1%
Dahbour 2017	4	10	40.0	[9.6; 70.4]	7.1%
Iacobaeus 2019	2	5	40.0	[0.0; 82.9]	3.5%
Harris 2018	10	20	50.0	[28.1; 71.9]	13.6%
Bonab 2012	13	22	59.1	[38.5; 79.6]	15.5%
		**127**			
**Random effects model**			**37.4**	**[29.3; 45.5]**	**100.0%**

Heterogeneity: I^2 = 0%, τ^2 = 0, χ^2_{11} = 10.74 (p = 0.47)

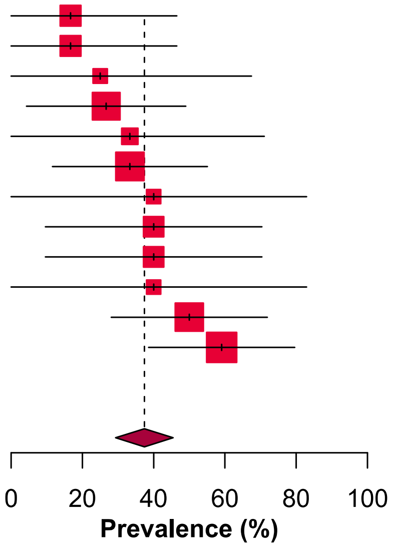

U

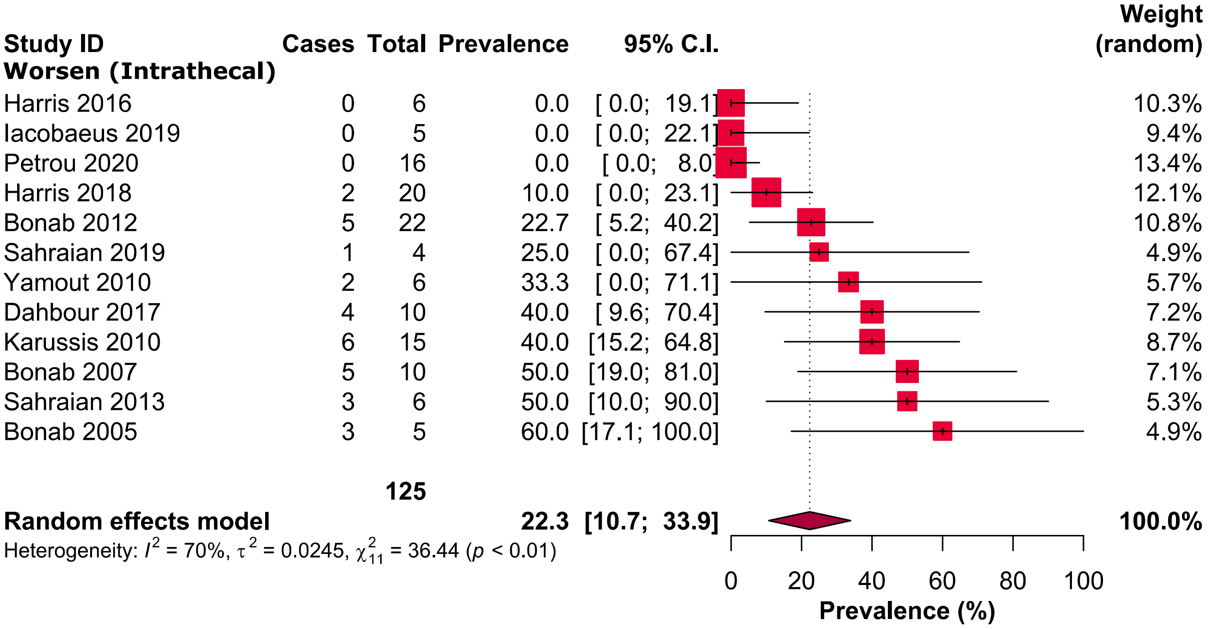

Figure S2. Subgroup analyses on improved, stable, and worsen after follow-up of ≤6 months (A-C), follow-up of >6 to 12 months (D-F) and follow-up of >12 months (G-I); bone marrow-derived stem cells (J-L) and umbilical cord or placenta-derived stem cells (M-O); and intravenous administration (P-R) and intrathecal administration (S-U) in multiple sclerosis patients following mesenchymal stem cell therapy.

A

Study ID	Cases	Total	Prevalence	95% C.I.	Weight (random)
**Improved multiple sclerosis patients**					
Bonab 2005	0	5	0.0	[0.0; 22.1]	5.3%
Bonab 2007	1	10	10.0	[0.0; 28.6]	5.7%
Bonab 2012	4	22	18.2	[2.1; 34.3]	6.0%
Cohen 2023	3	16	18.8	[0.0; 37.9]	5.7%
Dahbour 2017	2	10	20.0	[0.0; 44.8]	4.9%
De oliveira 2015	10	40	25.0	[11.6; 38.4]	6.4%
Karussis 2010	5	15	33.3	[9.5; 57.2]	5.1%
Shahraian 2013	2	6	33.3	[0.0; 71.1]	3.5%
Harris 2021	7	18	38.9	[16.4; 61.4]	5.2%
Harris 2018	8	20	40.0	[18.5; 61.5]	5.4%
Lublin 2014	5	12	41.7	[13.8; 69.6]	4.6%
Fernandez 2018	4	9	44.4	[12.0; 76.9]	4.0%
Meng 2018	1	2	50.0	[0.0; 100.0]	1.6%
Sahraian 2019	2	4	50.0	[1.0; 99.0]	2.6%
Yamout 2010	3	6	50.0	[10.0; 90.0]	3.3%
Petrou 2020	17	32	53.1	[35.8; 70.4]	5.9%
Iacobaeus 2019	3	5	60.0	[17.1; 100.0]	3.0%
Harris 2016	4	6	66.7	[28.9; 100.0]	3.5%
Cohen 2017	17	24	70.8	[52.6; 89.0]	5.8%
Petrou 2021	5	7	71.4	[38.0; 100.0]	3.9%
Lu 2013	6	8	75.0	[45.0; 100.0]	4.3%
Odinak 2012	6	8	75.0	[45.0; 100.0]	4.3%
		**285**			
**Random effects model**			**40.4**	**[30.6; 50.2]**	**100.0%**

Heterogeneity: $I^2 = 70\%$, $\tau^2 = 0.0348$, $\chi^2_{21} = 70.20$ ($p < 0.01$)

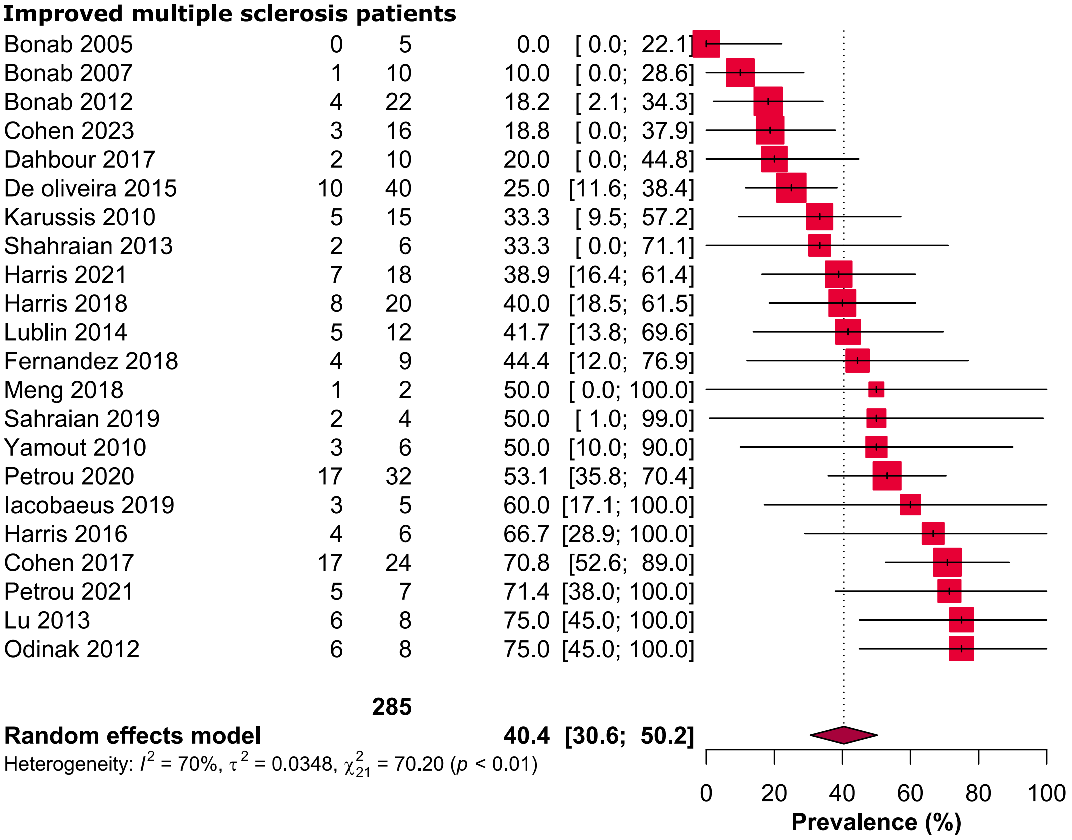

B

Study ID	Cases	Total	Prevalence	95% C.I.	Weight (random)
**Stable multiple sclerosis patients**					
Lu 2013	1	8	12.5	[0.0; 35.4]	5.7%
Odinak 2012	1	8	12.5	[0.0; 35.4]	5.7%
Cohen 2017	4	24	16.7	[1.8; 31.6]	8.4%
Shahraian 2013	1	6	16.7	[0.0; 46.5]	4.1%
Yamout 2010	1	6	16.7	[0.0; 46.5]	4.1%
Fernandez 2018	2	9	22.2	[0.0; 49.4]	4.6%
Sahraian 2019	1	4	25.0	[0.0; 67.4]	2.4%
Karussis 2010	4	15	26.7	[4.3; 49.0]	5.8%
Petrou 2021	2	7	28.6	[0.0; 62.0]	3.5%
Harris 2016	2	6	33.3	[0.0; 71.1]	2.9%
Lublin 2014	4	12	33.3	[6.7; 60.0]	4.8%
Harris 2021	6	18	33.3	[11.6; 55.1]	6.0%
Petrou 2020	12	32	37.5	[20.7; 54.3]	7.7%
Bonab 2005	2	5	40.0	[0.0; 82.9]	2.4%
Bonab 2007	4	10	40.0	[9.6; 70.4]	4.0%
Dahbour 2017	4	10	40.0	[9.6; 70.4]	4.0%
Iacobaeus 2019	2	5	40.0	[0.0; 82.9]	2.4%
Harris 2018	10	20	50.0	[28.1; 71.9]	6.0%
Meng 2018	1	2	50.0	[0.0; 100.0]	1.0%
Bonab 2012	12	22	54.5	[33.7; 75.4]	6.3%
De oliveira 2015	24	40	60.0	[44.8; 75.2]	8.3%
		**269**			
**Random effects model**			**32.8**	**[25.5; 40.1]**	**100.0%**

Heterogeneity: $I^2 = 41\%$, $\tau^2 = 0.0110$, $\chi^2_{20} = 33.83$ ($p = 0.03$)

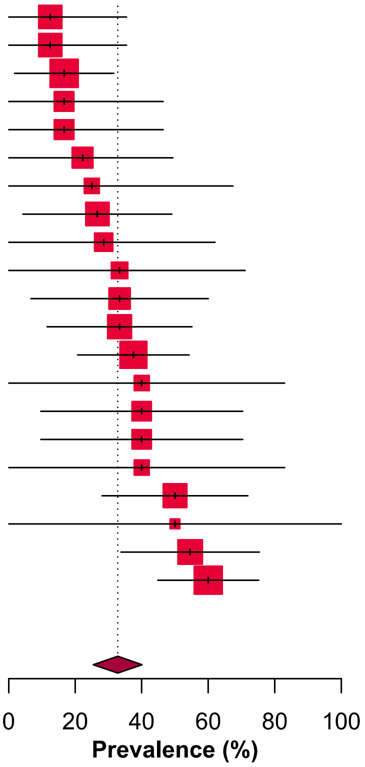

C

Study ID	Cases	Total	Prevalence	95% C.I.	Weight (random)
**Worsen multiple sclerosis patients**					
Harris 2016	0	6	0.0	[0.0; 19.1]	5.8%
Iacobaeus 2019	0	5	0.0	[0.0; 22.1]	4.8%
Meng 2018	0	2	0.0	[0.0; 42.2]	1.8%
Petrou 2021	0	7	0.0	[0.0; 16.8]	6.6%
Petrou 2020	3	32	9.4	[0.0; 19.5]	9.7%
Harris 2018	2	20	10.0	[0.0; 23.1]	8.2%
Cohen 2017	3	24	12.5	[0.0; 25.7]	8.2%
Lu 2013	1	8	12.5	[0.0; 35.4]	4.6%
Odinak 2012	1	8	12.5	[0.0; 35.4]	4.6%
De oliveira 2015	6	40	15.0	[3.9; 26.1]	9.2%
Lublin 2014	3	12	25.0	[0.5; 49.5]	4.2%
Sahraian 2019	1	4	25.0	[0.0; 67.4]	1.8%
Bonab 2012	6	22	27.3	[8.7; 45.9]	5.9%
Harris 2021	5	18	27.8	[7.1; 48.5]	5.3%
Fernandez 2018	3	9	33.3	[2.5; 64.1]	3.0%
Yamout 2010	2	6	33.3	[0.0; 71.1]	2.2%
Dahbour 2017	4	10	40.0	[9.6; 70.4]	3.1%
Karussis 2010	6	15	40.0	[15.2; 64.8]	4.2%
Bonab 2007	5	10	50.0	[19.0; 81.0]	3.0%
Shahraian 2013	3	6	50.0	[10.0; 90.0]	2.0%
Bonab 2005	3	5	60.0	[17.1; 100.0]	1.8%
		**269**			
**Random effects model**			**18.1**	**[12.0; 24.2]**	**100.0%**

Heterogeneity: I^2 = 42%, τ^2 = 0.0073, χ^2_{20} = 34.45 (p = 0.02)

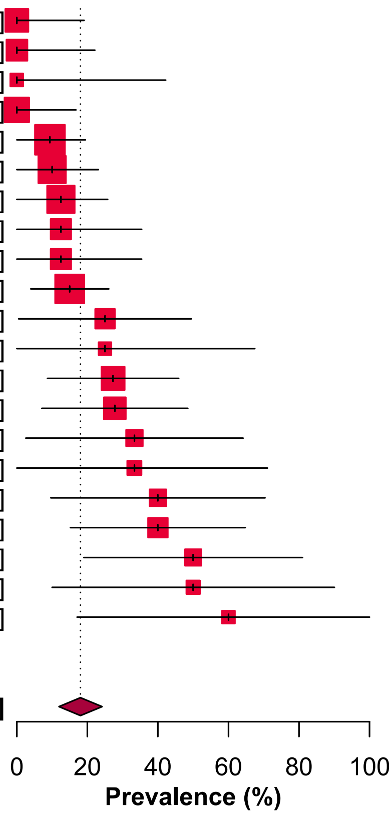

D

Study ID	Cases	Total	Prevalence	95% C.I.	Weight (random)
**Improved (excluding small studies)**					
Bonab 2007	1	10	10.0	[0.0; 28.6]	9.5%
Bonab 2012	4	22	18.2	[2.1; 34.3]	10.2%
Cohen 2023	3	16	18.8	[0.0; 37.9]	9.4%
Dahbour 2017	2	10	20.0	[0.0; 44.8]	7.9%
De oliveira 2015	10	40	25.0	[11.6; 38.4]	10.9%
Karussis 2010	5	15	33.3	[9.5; 57.2]	8.2%
Harris 2021	7	18	38.9	[16.4; 61.4]	8.5%
Harris 2018	8	20	40.0	[18.5; 61.5]	8.8%
Lublin 2014	5	12	41.7	[13.8; 69.6]	7.2%
Petrou 2020	17	32	53.1	[35.8; 70.4]	9.9%
Cohen 2017	17	24	70.8	[52.6; 89.0]	9.6%
		**219**			
**Random effects model**			**33.5**	**[22.2; 44.7]**	**100.0%**

Heterogeneity: I^2 = 73%, τ^2 = 0.0256, χ^2_{10} = 36.55 (p < 0.01)

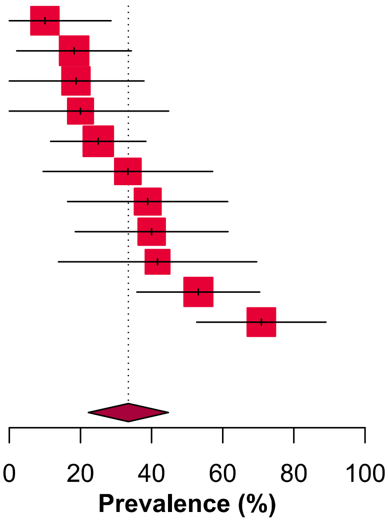

E

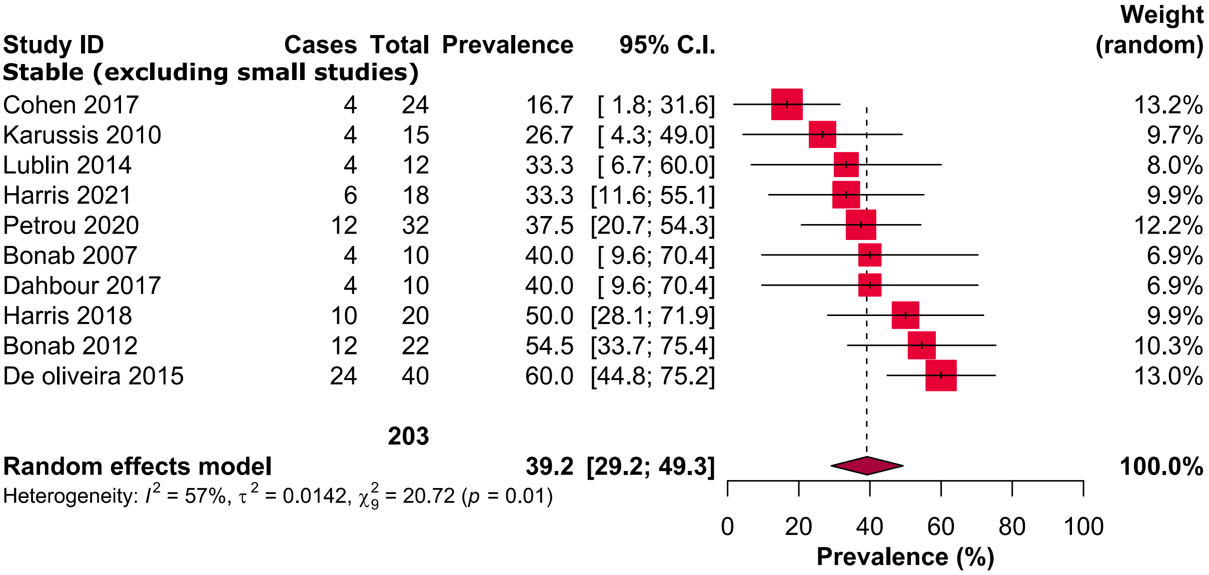

F

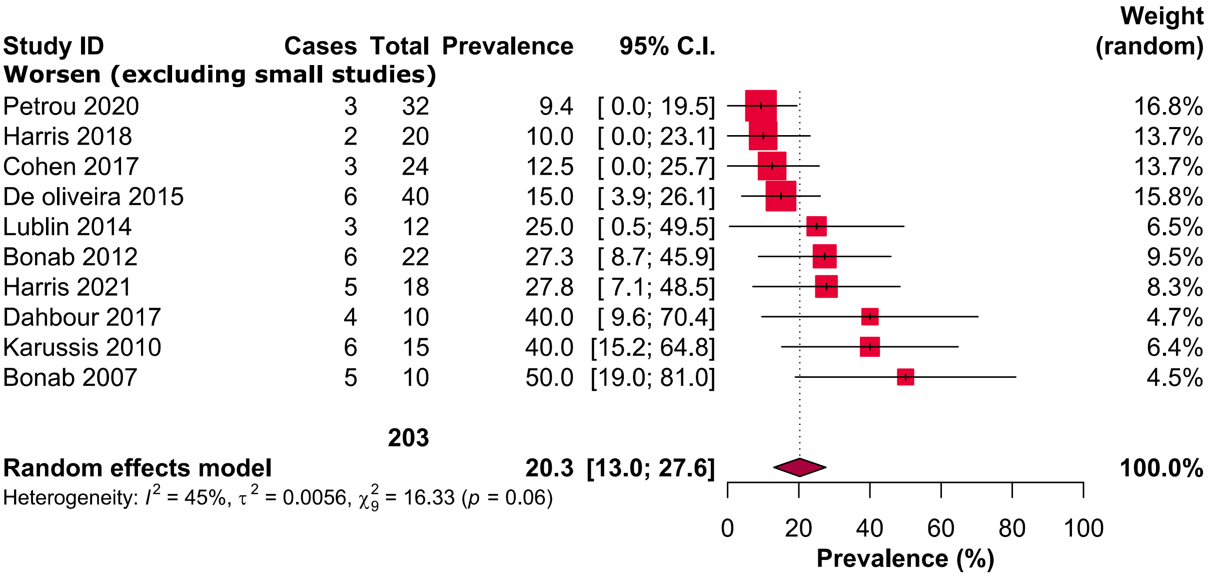

Figure S3. Sensitivity analyses assessing the effectiveness of mesenchymal stem cell therapy in patients with multiple sclerosis excluding low-quality studies (A-C) and excluding small studies (D-F).
